# PredictEFC: a fast and efficient multi-label classifier for predicting enzyme family classes

**DOI:** 10.1186/s12859-024-05665-1

**Published:** 2024-01-30

**Authors:** Lei Chen, Chenyu Zhang, Jing Xu

**Affiliations:** College of Information Engineering, Shanghai Maritime University, Shanghai, 201306 People’s Republic of China

**Keywords:** Enzymes, Family class, Multi-label classification, Functional domain, Dimension reduction, Random forest

## Abstract

**Background:**

Enzymes play an irreplaceable and important role in maintaining the lives of living organisms. The Enzyme Commission (EC) number of an enzyme indicates its essential functions. Correct identification of the first digit (family class) of the EC number for a given enzyme is a hot topic in the past twenty years. Several previous methods adopted functional domain composition to represent enzymes. However, it would lead to dimension disaster, thereby reducing the efficiency of the methods. On the other hand, most previous methods can only deal with enzymes belonging to one family class. In fact, several enzymes belong to two or more family classes.

**Results:**

In this study, a fast and efficient multi-label classifier, named PredictEFC, was designed. To construct this classifier, a novel feature extraction scheme was designed for processing functional domain information of enzymes, which counting the distribution of each functional domain entry across seven family classes in the training dataset. Based on this scheme, each training or test enzyme was encoded into a 7-dimenion vector by fusing its functional domain information and above statistical results. Random k-labelsets (RAKEL) was adopted to build the classifier, where random forest was selected as the base classification algorithm. The two tenfold cross-validation results on the training dataset shown that the accuracy of PredictEFC can reach 0.8493 and 0.8370. The independent test on two datasets indicated the accuracy values of 0.9118 and 0.8777.

**Conclusion:**

The performance of PredictEFC was slightly lower than the classifier directly using functional domain composition. However, its efficiency was sharply improved. The running time was less than one-tenth of the time of the classifier directly using functional domain composition. In additional, the utility of PredictEFC was superior to the classifiers using traditional dimensionality reduction methods and some previous methods, and this classifier can be transplanted for predicting enzyme family classes of other species. Finally, a web-server available at http://124.221.158.221/ was set up for easy usage.

**Supplementary Information:**

The online version contains supplementary material available at 10.1186/s12859-024-05665-1.

## Introduction

Enzymes, also named biocatalysts, are a special type of proteins, which can speed up cellular biochemical processes. It is known that the energy to maintain the living organisms is produced by various chemical reactions. Almost all these reactions need enzymes to participate in. Thus, enzymes are the essential matters for living organisms. With the accumulation of the knowledge on enzymes, our understanding on them has been sharply improved. To distinguish enzymes with different functions, each enzyme was assigned at least one Enzyme Commission (EC) number. Such number is composed of four digits, such as 1.1.1.1. According to mechanisms of catalytic reactions, enzymes can be classified into seven family classes: (1) Oxidativereductases; (2) Transferases; (3) Hydrolases; (4) Lyases; (5) Isomerases; (6) Ligases and (7) Translocases, which are represented by the first digit of enzyme EC numbers. This enzyme classification is recommended by Nomenclature Committee of the International Union of Biochemistry and Molecular Biology (IUBMB, https://iubmb.qmul.ac.uk/). Identification of the family classes of enzymes is the first step to uncover its functions.

Traditional methods to identify the family classes of enzymes needs lots of costs and time. In the past twenty years, several computational methods have been proposed to predict the family classes or EC numbers of enzymes, providing an alternative way to investigate enzymes. Most of them are machine learning based methods. Some methods were proposed to predict the first digit of enzyme EC numbers (i.e., the family classes of enzymes) [1–3]. More methods were designed to first predict enzyme or non-enzyme, and then to recognize the family classes of enzymes [4–13]. To obtain the entire EC numbers of enzymes, some methods were built to identify the second digit of EC numbers (i.e., the sub-classes of family classes of enzymes) [14–18]. However, these methods cannot predict enzyme or non-enzyme and the family classes of enzymes. An important top-down approach, proposed by Shen and Chou, integrated above methods by first determining enzyme or non-enzyme and them identifying the first two EC numbers of enzymes [19]. A recent method, UDSMProt [20], was also designed for this purpose. The later methods are devoted to predicting entire EC numbers of enzymes, such as BENZ WS [21], ECPred [22], DEEPre [23], and EFICAz^2.5^ [24]. Extraction of informative features from enzymes is an important step for designing most above methods. Popular enzyme features include amino acid composition [4, 14], pseudo amino acid composition [1, 5–7, 15–18], protein structure [2, 3, 8, 9, 23], functional domain composition [10, 11, 13, 18, 19], gene ontology [6], pseudo position-specific scoring matrix [12, 13, 19, 23], physicochemical properties [22, 23]. Selection of proper classification algorithms is another important step to build the efficient machine learning based methods. Several algorithms have been adopted, such as artificial neuron network (ANN) [25], support vector machine (SVM) [1, 2, 4, 7, 11, 16], Bayesian [3], nearest neighbor algorithm (NNA) [6, 10, 17, 19], linear discriminant analysis (LDA) [8, 9], hidden Markov model [21], ensemble learning [22, 24], and deep learning algorithms [20, 23]. When investigating the family classes of enzymes, all methods, except BENZ WS [21], considered six family classes of enzymes. However, in 2018, IUBMB added the seventh family class (translocases), inducing the limitations on applications of these methods. It is necessary to reconstruct efficient computational methods to predict family classes of enzymes, even the EC numbers of enzymes. On the other hand, few previous methods can deal with enzymes belonging to multiple family classes. In fact, several enzymes can belong to two or more family classes. The methods in [12, 13] can identify multiple family classes of enzymes. However, they cannot identify the seventh enzyme family class (translocases). Thus, the multi-label classifiers for prediction of family classes of enzymes are still needed. As mentioned above, functional domain composition is an important feature type to describe enzymes and the methods with such representation always provide good performance. However, such representation always involves a problem of dimension disaster, i.e., each enzyme is represented by a large number of features, which reduces the efficiency of the classifiers based on this representation. This study was conducted with the above background. We want to design an effective feature extraction scheme from functional domain information and build a multi-label classifier to predict family classes of enzymes with high performance and efficiency.

In this study, a multi-label classifier was proposed for identifying family classes of enzymes. Enzymes were represented by features extracted from their functional domains. To avoid dimension disaster, a novel feature extraction scheme was designed, which conducted a deep analysis on each involved functional domain entry across all enzyme family classes in the training dataset. The analysis result was used to encode the test or training enzyme in terms of its functional domain information, yielding a low-dimension feature representation for each enzyme. With such representation of enzymes, random k-labelsets (RAKEL) [26, 27] was employed to build the multi-label classifier, named PredictEFC, where random forest (RF) [28] was selected as the base classification algorithm. The tenfold cross-validation results on the benchmark dataset shown that the accuracy and absolute true were 0.8493 and 0.8350, respectively, indicating the good performance of the classifier. Its performance on two independent datasets was also high. The accuracy values reached 0.9118 and 0.8777, which were higher than those yielded by two previous methods. Although the PredictEFC was a little weaker than the classifier directly using functional domain composition, its efficiency was largely improved, indicating the utility of the feature extraction scheme. Furthermore, PredictEFC outperformed the classifiers using enzyme features that were obtained by applying popular dimensionality reduction methods on functional domain composition.

## Materials and methods

### Benchmark dataset

A rigorous and objective benchmark dataset is the base for developing efficient classifiers. Although several datasets on enzymes have been proposed in the past twenty years [29], they were not complete enough as new discoveries have been added in recent years, especial for the addition of the seventh family class in 2018. Thus, we downloaded 3550 human enzymes and their EC numbers from Expasy (https://enzyme.expasy.org/, accessed in August 2022), a repository of information on the nomenclature of enzymes. All enzymes were represented by their UniProt IDs. Based on these IDs, the protein sequences were retrieved from UniProt. As the homogenous proteins may overestimate the constructed classifiers, the well-known tool, CD-HIT [30], was adopted to exclude proteins with similar sequences. 2382 enzymes remained, which constituted the benchmark dataset of this study, denoted by *S*. The sequence identity of any two proteins in this dataset was less than 0.4. According to the first codes of the EC numbers of these enzymes, 2382 enzymes were classified into seven family classes, which are listed in the second column of Table 1. For convenience, seven family classes were tagged as EC 1–7. Let us denote the enzyme set consisting of enzymes in EC *i* as *S*_*i*_ (1 ≤ *i* ≤ 7). Then, the benchmark dataset *S* can be formulated by

**Table 1:** Breakdown of the enzymes in the benchmark dataset and two independent datasets

Tag	Enzyme family class	Number of enzymes		
		Benchmark dataset	Independent dataset I	Independent dataset II
EC 1	Oxidoreductases	288	5	0
EC 2	Transferases	1080	13	57
EC 3	Hydrolases	747	14	60
EC 4	Lyases	113	0	5
EC 5	Isomerases	80	0	8
EC 6	Ligases	86	0	6
EC 7	Translocases	51	2	3
Sum		2445	34	139
Number of different enzymes		2382	34	139
The multiplicity degree		1.026	1	1

1 $$S= {S}_{1}\cup {S}_{2}\cup {S}_{3}\cup {S}_{4}\cup {S}_{5}\cup {S}_{6}\cup {S}_{7}$$

Enzymes, denoted by UniProt IDs, in each set are provided in Additional file 1. The number of enzymes in each *S*_*i*_ was counted and is also listed in Table 1. As some enzymes can belong to more than one family class, the sum of above numbers (2445) was larger than the number of different enzyme (2382). Evidently, it was a multi-label classification problem for assigning family classes to enzymes.

**Fig. 1:**
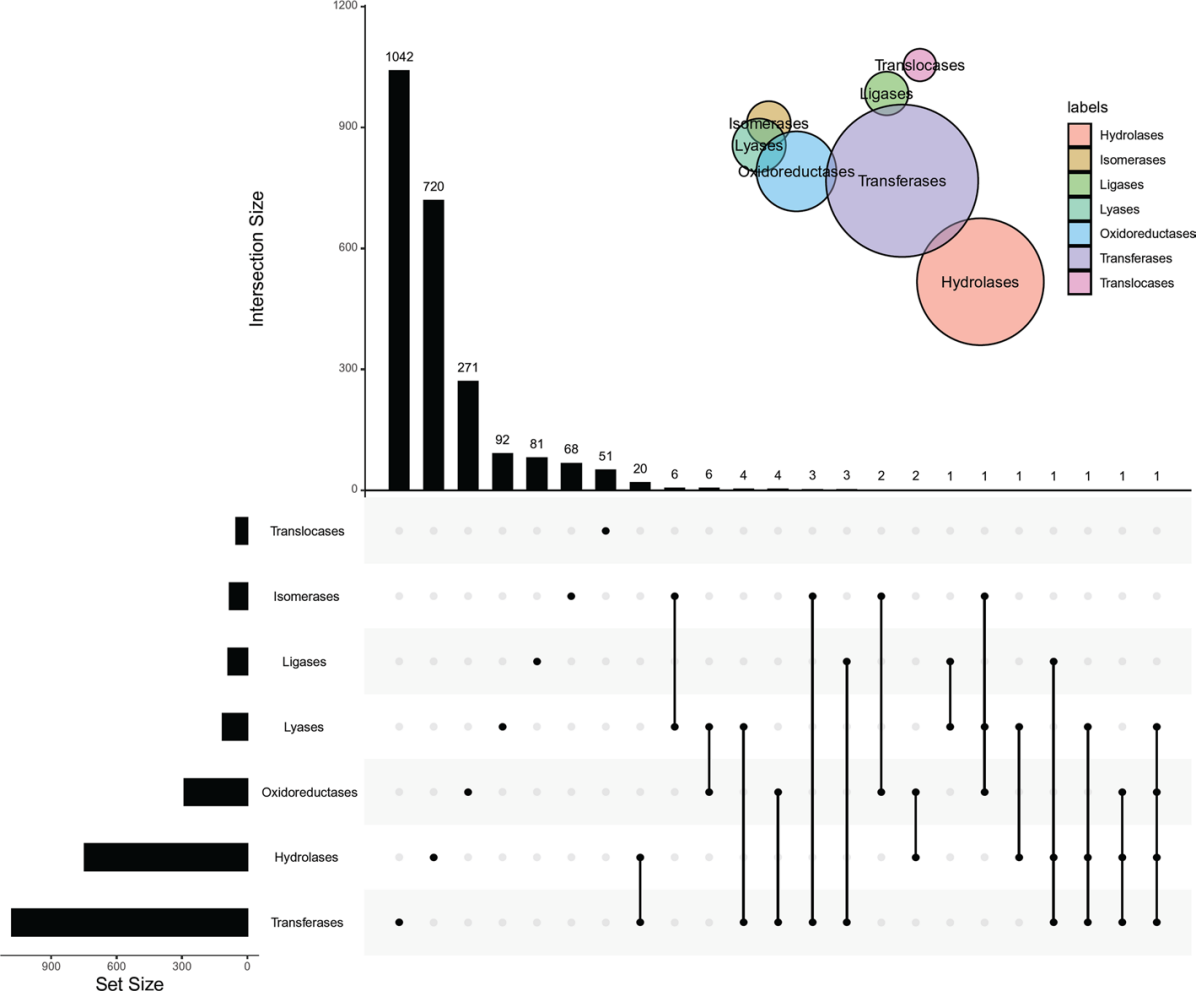
Upset graph to show the distribution of enzymes across seven family classes. Some classes share common enzymes, meaning that some enzymes belong to more than one family classes

For multi-label classification problems, it is necessary to count the multiplicity degree (MD), which is defined as the average number of labels for samples. For the benchmark dataset *S*, MD was 1.026 (2445/2382), meaning that each enzyme belongs to 1.026 family classes. An upset graph was plotted to show the intersection of enzymes in seven family classes, as illustrated in Fig. 1. It can be observed that hydrolases and transferases shared 20 common enzymes, isomerases and lyases had six common enzymes. Few enzymes belong to more than two family classes.

Two independent datasets were constructed for testing the generalization ability of PredictEFC. The first independent dataset was also retrieved from Expasy. The latest information of human enzymes and their EC numbers were extracted (Accessed in November 2023), obtaining 3593 enzymes. We constructed the independent dataset from these enzymes using the following procedures: (1) These enzymes were combined with 2382 enzymes to constitute a large enzyme dataset, which was processed by CD-HIT with cutoff 0.4; (2) The 2382 enzymes were excluded; (3) Remaining enzymes without functional domain information were discarded. 34 enzymes were obtained under above operations, which comprised the first independent dataset, called independent dataset I. The second independent dataset was extracted from Kyoto Encyclopedia of Genes and Genomes (KEGG) ENZYME [31] (Accessed in November 2023). Obtained enzymes were processed with the similar data cleaning procedures for the independent dataset I. Finally, 139 enzymes and their EC numbers were obtained, which constituted the second independent dataset, called independent dataset II. The breakdown of these two independent datasets is also provided in Table 1 and the detailed enzymes in them are provided in Additional files 2 and 3.

### Enzyme representation

In machine learning, sample representation is an important step. A good representation should contain essential properties of samples as complete as possible. As mentioned in Sect. "Introduction", the functional domain information is useful materials to indicate the essential properties of proteins, from which informative features can be extracted. In this study, such information was employed as the raw data for extracting protein features. A novel feature extraction scheme was designed to extract informative features of enzymes.

The functional domain information of 2382 enzymes was retrieved from the InterPro database (http://ftp.ebi.ac.uk/pub/databases/interpro/, version 88.0, accessed in March 2022) [32, 33]. 5117 IPR terms were involved for 2382 enzymes. The average number of IPR terms for one enzyme was 5.66. For easy descriptions, the IPR terms of protein *p* constituted a set, denoted by *IPR*(*p*), which was formulated by

2 $$IPR\left(p\right)=\{{IPR}_{p}^{1},{IPR}_{p}^{2},\cdots ,{IPR}_{p}^{k}\}$$

where *k* was the number of IPR terms annotated to protein *p*.

Functional domain composition is a traditional way to use functional domain information for protein representation. In this way, a binary vector is built for each protein according to its IPR terms. Each component corresponds to one IPR term. It is set to 1 if such IPR term is annotated to the protein; otherwise, it is set to zero. Evidently, such representation could lead to the problem of dimension disaster as huge number of IPR terms have been defined to date. For enzymes investigated in this study, each of them could be represented by a 5117-dimensional binary vector. The efficiency of the classifiers based on such representation is not very high. In view of this, this study proposed a novel scheme to give a deep insight into the IPR terms of training enzymes and then assign informative features to a given enzyme.

Given a training enzyme dataset, denoted by ${S}_{tr}=\{{e}_{1},{e}_{2},\cdots ,{e}_{n}\}$, where *n* is the number of enzymes in ${S}_{tr}$, pick up all related IPR terms that is annotated to at least one enzyme. Suppose that there are *m* IPR terms, formulated by

3 $${IPR}^{1},{IPR}^{2},\cdots ,{IPR}^{m}$$

For each IPR term, say ${IPR}^{i}$, count the number of training enzymes that are annotated by ${IPR}^{i}$. Such term is denoted by $N({IPR}^{i})$, i.e.,

4 $$N\left({IPR}^{i}\right)=|\left\{e|e\in {S}_{tr}\ and\ {IPR}^{i}\in IPR(e)\right\}|$$

As the labels of training enzymes can be observed, all training enzymes can be divided into *L* subsets, where *L* is the number of different labels (*L* = 7 in this study), denoted by ${S}_{tr}^{j}$ (1 ≤ *j* ≤ *L*). For ${IPR}^{i}$, the second term was computed for each enzyme subset, denoted by ${N}_{j}\left({IPR}^{i}\right)$, which can be formulated by

5 $${N}_{j}\left({IPR}^{i}\right)=|\left\{e|e\in {S}_{tr}^{j}\ and\ {IPR}^{i}\in IPR(e)\right\}|$$

Cleary, ${N}_{j}\left({IPR}^{i}\right)$(1 ≤ *j* ≤ *L*) indicate the distribution of ${IPR}^{i}$ across *L* labels. Intuitively, if a protein annotated by ${IPR}^{i}$, it is more likely to be assigned the label whose ${N}_{j}\left({IPR}^{i}\right)$ is maximum. As the range of $N\left({IPR}^{i}\right)$ and ${N}_{j}\left({IPR}^{i}\right)$(1 ≤ *j* ≤ *L*) greatly varies for different IPR terms, direct usage of $N\left({IPR}^{i}\right)$ and ${N}_{j}\left({IPR}^{i}\right)$ (1 ≤ *j* ≤ *L*) is not an excellent choice. In view of this, $N\left({IPR}^{i}\right)$ and ${N}_{j}\left({IPR}^{i}\right)$ are combined as

6 $${R}_{j}\left({IPR}^{i}\right)=\frac{{N}_{j}\left({IPR}^{i}\right)}{N\left({IPR}^{i}\right)}$$

${R}_{j}\left({IPR}^{i}\right)$ indicates the proportion of enzymes with the *j*-th label that are annotated by ${IPR}^{i}$ among all training enzymes annotated by the same IPR term. A large value means that such IPR term may be highly related to the *j*-th label, which can be further induced that enzymes annotated by such IPR term have the *j*-th label with a high probability. Furthermore, after such operation, ${R}_{j}\left({IPR}^{i}\right)$ is all between 0 and 1 no matter which label or IPR term is involved. These values are suitable raw materials to encode enzymes. On one hand, they contain the distribution information of IPR terms. On the other hand, the label information is also included. Above information is useful to determine the family classes of enzymes.

Given a training or test enzyme *p*, it can be encoded into a vector according to the above entries and its IPR terms. Suppose that its IPR terms are formulated by **Eq. **2. For each IPR term, say ${IPR}_{p}^{i}$ (1 ≤ *i* ≤ *k*), pick up ${R}_{j}\left({IPR}_{p}^{i}\right)$ (1 ≤ *j* ≤ *L*) calculated from the training dataset. For the *j*-th label, compute the following entry:

7 $${X}_{j}={\text{max}}\left\{{R}_{j}\left({IPR}_{p}^{1}\right),{R}_{j}\left({IPR}_{p}^{2}\right),\cdots ,{R}_{j}\left({IPR}_{p}^{k}\right)\right\}$$

${X}_{j}$ indicates the maximum proportion of enzymes with the *j*-th label among all enzymes across all IPR terms of the enzyme *p*. Generally, a high value suggests it is more likely for *p* to share the *j*-th label, which is helpful for making correct classification. As seven labels (family classes) were involved in this study, each enzyme can be encoded into a 7-dimension vector, as formulated by

8 $$V\left(p\right)={[{X}_{1}, {X}_{2}, {X}_{3}, {X}_{4},{ X}_{5}, {X}_{6},{X}_{7}]}^{T}$$

Compared with the binary vector obtained by functional domain composition, the dimension is sharply reduced, which give a strong base for building classifiers with high efficiency.

### Classifier construction

As mentioned in Sect. "Benchmark Dataset", some enzymes can belong to more than one family class. A multi-label classifier should be designed to assign family classes to the test enzyme. In multi-label machine learning, problem transformation is a widely used scheme to design multi-label classifiers. Such scheme transforms the original problem into multiple single-label classification problems [34]. In this study, we adopted such scheme to design the multi-label classifier.

Label Powerset (LP) is a classic problem transformation scheme in multi-label learning. This method takes the members of the powerset of label set as new labels and assigns a new label to each sample according to its original labels. Under such operation, each sample has exact one label. A single-label classifier can be built based on samples that has been assigned new labels. The LP method has an evident defect. With the raising of label number, the size of powerset sharply increases. It is indicated that lots of new labels are employed, inducing label disaster. Furthermore, some labels may have few samples, reducing the learning efficiency. To tackle such problem, its improved version, RAKEL [26, 27], was designed. This method employs the random selection of labels. If the classification problem containing *L* labels, it randomly selects* k* labels from all labels, where 1 ≤ *k* ≤ *L*. This procedure is executed multiple times to cover all labels, i.e., each label is selected at least once. Such number of times is determined by another parameter of RAKEL, denoted by *m*. Accordingly, *m* label subsets are constructed. On each subset, LP method is applied to set up a LP classifier with a given single-label classification algorithm. All *m* LP classifiers are integrated to build the final classifier.

As a problem transformation method, a single-label classification algorithm is necessary. In this study, we tried two classic classification algorithms: RF [28] and SVM [35]. RF is an ensemble algorithm consisting of several decision trees. Each tree is built by randomly selecting samples and features. For a test sample, each tree provides the prediction. These predictions are integrated with majority voting in RF. SVM is a powerful classification algorithm based on statistical theory. It tries to find out a hyperplane that can separate samples in two classes as perfect as possible. In many cases, samples are mapped into a high-dimensional space via a kernel function so that such hyperplane is easy to be discovered. For a test sample, it is also mapped into the same high-dimensional space and its prediction is determined according to the side of hyperplane it is located. RF and SVM have been widely used to tackle many biological problems [36–42]. The final classifier would select the classification algorithm that can provide the best performance.

To quickly implement RAKEL, the tool “RAKEL” in Meka (http://waikato.github.io/meka/, version 1.9.3) [43] was adopted in this study. The RF and SVM were also implemented by tools “RandomForest” and “SMO” in Meka. Some parameters of above tools were tuned to access the optimal multi-label classifier.

### Performance evaluation

Cross-validation is a commonly used method to evaluate the performance of classifiers [44]. Such method always equally and randomly divides samples into *K* parts. Each part is singled out one by one to constitute the test dataset, whereas the rest *K*-1 parts are used to constitute the training dataset. The classifier built on the training dataset is applied on the test dataset. The average performance on *K* parts is computed to assess the performance of the classifier. *K* is always set to five or ten. Here, it was set to 10, i.e., tenfold cross-validation was adopted to evaluate all classifiers in this study. It is necessary to point out that the enzyme representation is highly related to the training dataset. The representations for the same enzyme in different rounds of cross-validation are different. Thus, when executing tenfold cross-validation, we always divided the samples in advance and then generate the representations of enzymes. The above general cross-validation randomly divided samples into some parts, which may cause differences between the distributions of samples in the test dataset and those in the whole dataset. This problem can be reduced by employing stratified sampling. This study further adopted this method to construct test and training datasets in tenfold cross-validation. In details, we first divided enzymes into nine groups, where seven groups contained enzymes exactly belonging to seven family classes, the eighth group included the enzymes exactly belonging to hydrolases and transferases, and the last group consisted of the rest enzymes. Then, enzymes in each group were randomly divided into ten parts. Parts with the same index for nine groups were combined to constitute one fold of the cross-validation. The above sample partition procedures can guarantee that the distributions of enzymes in ten folds are similar, which are also similar to the distribution of samples in the whole dataset.

Several measurements have been proposed to evaluate the quality of predicted results of the multi-label classifiers. In this study, two sets of measurements were employed, where the first set was to assess the overall performance of the classifier and the second set can assess the performance of the classifier on different family classes. Five measurements contained in the first set include aiming, coverage, accuracy, absolute true, and absolute false [45–51]. They can be computed by

9 $$Aiming=\frac{1}{N}\sum_{k=1}^{N}\frac{\Vert {L}_{k}\cap {L}_{k}^{*}\Vert }{\Vert {L}_{k}^{*}\Vert },$$

10 $$coverage=\frac{1}{N}\sum_{k=1}^{N}\frac{\Vert {L}_{k}\cap {L}_{k}^{*}\Vert }{\Vert {L}_{k}\Vert },$$

11 $$accuracy=\frac{1}{N}\sum_{k=1}^{N}\frac{\Vert {L}_{k}\cap {L}_{k}^{*}\Vert }{\Vert {L}_{k}\cup {L}_{k}^{*}\Vert },$$

12 $$absolute\ true=\frac{1}{N}\sum_{k=1}^{N}\Delta ({L}_{k},{L}_{k}^{*}),$$

13 $$absolute\ false=\frac{1}{N}\sum_{k=1}^{N}\frac{\Vert {L}_{k}\cup {L}_{k}^{*}\Vert -\Vert {L}_{k}\cap {L}_{k}^{*}\Vert }{M},$$

where *N* denoted the number of enzymes, *M* represented the total number of labels (*M* = 7 in this study), ${L}_{k}$ indicated the subset of observed labels of the *k*-th enzyme, ${L}_{k}^{*}$ was the subset of predicted labels of the *k*-th enzyme, $\Delta ({L}_{k},{L}_{k}^{*})$ was determined by comparing ${L}_{k}$ and ${L}_{k}^{*}$, which can be calculated by

14 $$\Delta \left({L}_{k},{L}_{k}^{*}\right)=\left\{\begin{array}{cc}1,& if {L}_{k}\ is\ same\ as\ {L}_{k}^{*} \\ 0,& otherwise\end{array}\right.$$

Among these five measurements, the higher the aiming, coverage, absolute true and accuracy, the higher  the performance of the classifier. Absolute false is on the contrary. A low value suggests the high performance.

Measurements in the second set assess the classifier’s performance on different family classes. To compute these measurements, the true positive (TP), false positive (FP), true negative (TN) and false negative (FN) for one family class should be defined in advance. Take the *i-*th family class as an example. Enzymes in this class are termed as positive samples, whereas other enzymes are regarded as negative samples. Then, TP, FP, TN and FN can be defined as their definitions in binary classification. Accordingly, the following five measurements: accuracy, recall, precision, F1-measure and Matthews correlation coefficient (MCC) [52, 53], can be computed based on them, formulated by

15 $$accuracy\left(i\right)=\frac{TP\left(i\right)+TN\left(i\right)}{TP\left(i\right)+TN\left(i\right)+FP\left(i\right)+FN\left(i\right)}$$

16 $$recall\left(i\right)=\frac{TP\left(i\right)}{TP\left(i\right)+FN\left(i\right)}$$

17 $$precision\left(i\right)=\frac{TP\left(i\right)}{TP\left(i\right)+FP\left(i\right)}$$

18 $$F1-measure\left(i\right)=\frac{2\times TP\left(i\right)}{2\times TP\left(i\right)+FP\left(i\right)+FN(i)}$$

19 $$MCC\left(i\right)=\frac{TP\left(i\right)\times TN\left(i\right)-FP(i)\times FN(i)}{\sqrt{(TP\left(i\right)+FP(i))\times (TP\left(i\right)+FN(i))\times (TN\left(i\right)+FP(i))\times (TN\left(i\right)+FN(i))}}$$

In addition to these four measurements, the ROC and PR curves were also employed to fully display the performance of the classifier on different family classes. The areas under these two curves, denoted by AUROC and AUPR, were also calculated to show the performance of the classifier on one family class.

## Results and discussion

**Fig. 2:**
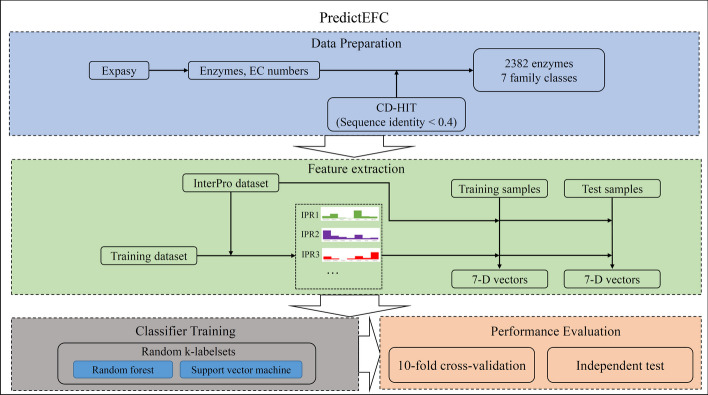
Entire construction and evaluation procedures of PredictEFC. The enzymes and their EC numbers are retrieved from Expasy. These enzymes are processed by CD-HIT to access a high-quality enzyme dataset, involving 2382 enzymes and 7 family classes. From the training dataset, the distribution of each functional domain (IPR term) is counted, which is used to encode training and test samples into 7-D vectors. The vectors are fed into random k-labelsets, with random forest or support vector machine as the base classification algorithm, for training the classifier. The classifier is assessed by tenfold cross-validation and independent test

In this study, a new multi-label classifier, PredictEFC, was designed to identify family classes of enzymes, which adopted the compact features derived from proteins’ functional domain information via a novel feature extraction scheme. The entire construction and evaluation procedures are illustrated in Fig. 2.

### Parameter selection

There were some parameters in the proposed multi-label classifier, PredictEFC, which should be tuned. For RAKEL, the parameter *k* was set to 3, 5, and 7, whereas the other parameter *m* was set to its default value of 10. The classification algorithm was set to RF or SVM. For RF, its main parameter *I*, number of decision trees, was set to various values in [80, 500]. As for SVM, the regularization parameter *C* was set to 0.5, 1, 3, 5, and 7, whereas four kernel functions were attempted, including polynomial kernel, normalized polynomial kernel, Puk kernel and RBF kernel. Their parameters were set as follows. Polynomial kernel: exponent *e* was set to various values in [1, 3]. Normalized polynomial kernel: exponent *e* was set to various values in [1, 3].

Puk kernel: default setting in Meka.

RBF kernel: parameter γ was set to various values in [0.1, 3.0].

As mentioned above, each parameter was set to several values in a certain scope. We adopted grid research to construct classifiers with all possible parameter combinations. According to the evaluation results yielded by tenfold cross-validation, the optimal values for each parameter can be determined and the classifier with optimal parameters were built as PredictEFC.

### Performance of the PredictEFC

According to Sect. "Parameter selection", several multi-label classifiers with all possible parameter combinations, yielded by grid research, were constructed. These classifiers were evaluated by tenfold cross-validation. With different base classification algorithms (SVMs with different kernels were deemed to be different), the best tenfold cross-validation results, measured by accuracy, are listed in Table 2. It can be observed that all classifiers seems to give similar performance. In detail, accuracy was around 0.8450, absolute true was about 0.8350, aiming was between 0.85 and 0.86 and coverage was between 0.84 and 0.87. The absolute false was around 0.0450. Evidently, such performance was quite high. Among these classifiers, the classifier using RF as the base classification algorithm was relatively better than others as it provided the best absolute false, aiming and accuracy, whereas the absolute true and coverage were ranked at the second place. Thus, we set this classifier as the proposed multi-label classifier, PredictEFC.

**Table 2:** Performance of the multi-label classifiers using different base classification algorithms

Base classification algorithm	Parameter	Absolute false	Absolute true	Aiming	Coverage	Accuracy	Time(s)
Random forest	*k* = 7, *I* = 500	**0.0444**	0.8350	**0.8577**	0.8563	**0.8493**	716.09
Support vector machine (Polynomial kernel)	*k* = 7, *C* = 1, *e* = 1	0.0460	0.8329	0.8522	0.8422	0.8422	**628.99**
Support vector machine (Normalized polynomial kernel)	*k* = 5, *C* = 0.5, *e* = 2	0.0454	0.8283	0.8573	**0.8604**	0.8483	636.80
Support vector machine (Puk kernel)	*k* = 7, *C* = 5	0.0461	0.8325	0.8530	0.8447	0.8430	1278.65
Support vector machine (RBF kernel)	*k* = 7, *C* = 7, γ = 0.1	0.0450	**0.8363**	0.8556	0.8456	0.8456	1368.10

The general tenfold cross-validation adopted the random division of samples, causing the different performance of the classifier on different folds. Table 3 displays the detailed performance of PredictEFC on ten folds. It can be observed that the standard deviations for five measurements were quite small, indicating that the performance of PredictEFC on ten folds was quite similar.

**Table 3:** Detailed cross-validation results of PredictEFC

Fold	Absolute false	Absolute true	Aiming	Coverage	Accuracy
1	0.0406	0.8410	0.8696	0.8682	0.8591
2	0.0389	0.8619	0.8703	0.8787	0.8703
3	0.0486	0.8235	0.8424	0.8361	0.8340
4	**0.0336**	**0.8739**	**0.8887**	**0.8845**	**0.8824**
5	0.0432	0.8361	0.8739	0.8666	0.8582
6	0.0408	0.8445	0.8718	0.8782	0.8634
7	0.0522	0.8067	0.8323	0.8361	0.8239
8	0.0570	0.7773	0.8169	0.8256	0.8064
9	0.0468	0.8277	0.8529	0.8466	0.8424
10	0.0486	0.8151	0.8375	0.8424	0.8312
Standard deviation	0.0069	0.0277	0.0227	0.0213	0.0235

**Fig. 3:**
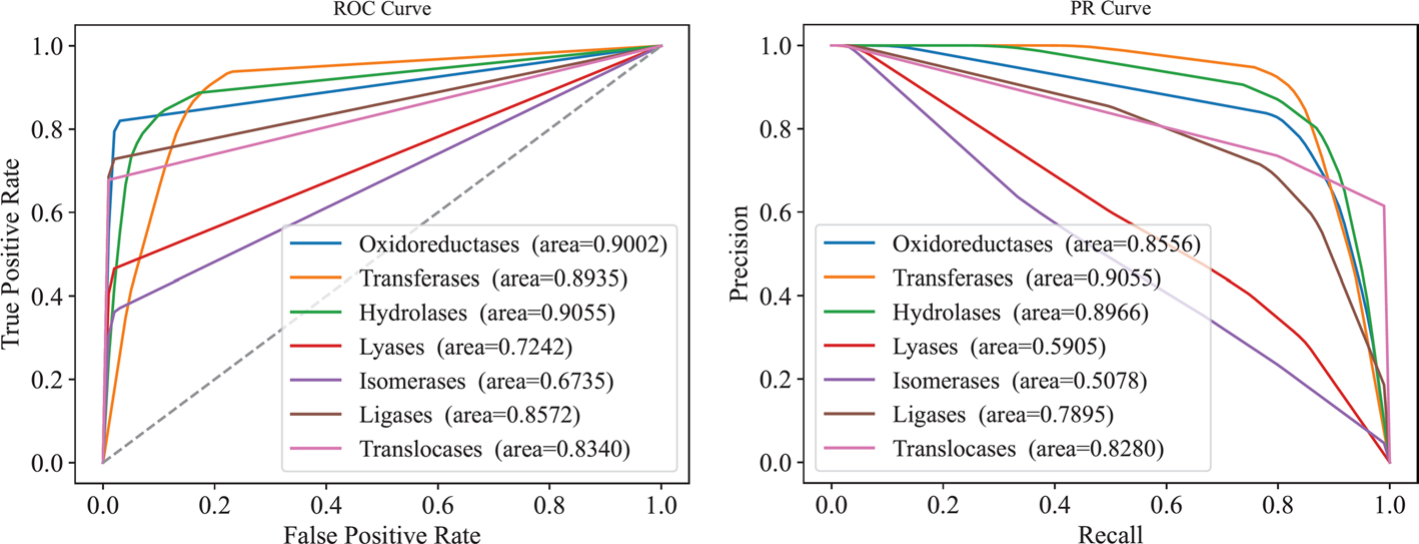
ROC and PR curves of PredictEFC for seven family classes. **A** ROC curves on seven family classes; **B** PR curves on seven family classes

To fully evaluate the performance of PredictEFC, we further calculated the accuracy, precision, recall, F1-measure, and MCC on seven family classes, which are listed in Table 4. The accuracies were all very high (≥ 0.89). For precision, PredictEFC provided high performance on five classes (oxidoreductases, transferases, hydrolases, ligases, and translocases). The recall, F1-measure, and MCC values were high for four classes (oxidoreductases, transferases, hydrolases and ligases). In addition, the ROC and PR curves were plotted for each family class, as shown in Fig. 3. The AUROC and AUPR were also listed in this figure and Table 4. Evidently, the AUROC and AUPR values were high for three classes (oxidoreductases, transferases, and hydrolases). However, those on lyases and isomerases were low. In generally, the PredictEFC provided satisfied performance for the classification of enzymes.

**Table 4:** Performance of the PredictEFC on seven family classes

Family class	Accuracy (*i*)	Precision (*i*)	Recall (*i*)	F1-measure(*i*)	MCC (*i*)	AUROC (*i*)	AUPR (*i*)
Oxidoreductases	0.9652	0.8726	0.8173	0.8424	**0.8240**	0.9002	0.8556
Transferases	0.8913	0.8502	**0.9291**	**0.8856**	0.7899	0.8937	**0.9055**
Hydrolases	0.9198	0.8827	0.8704	0.8730	0.8207	**0.9055**	0.8966
Lyases	0.9664	0.6973	0.4590	0.5454	0.5489	0.7242	0.5905
Isomerases	0.9698	0.5375	0.3570	0.4146	0.4225	0.6735	0.5078
Ligases	0.9841	0.8462	0.7234	0.7465	0.7750	0.8572	0.7895
Translocases	**0.9874**	**1.0000**	0.4000	0.5714	0.6283	0.8340	0.8280

Furthermore, the PredictEFC was tested by the tenfold cross-validation under stratified sampling. The detailed performance is listed in Table 5. It can be found that the absolute false, absolute true, aiming, coverage, and accuracy were 0.0481, 0.8207, 0.8449, 0.8463, and 0.8370, respectively. This performance was slightly lower than that yielded by the general tenfold cross-validation. The key measurements: accuracy and absolute true were 0.0123 and 0.0143 lower, respectively. This gap can be concluded that the performance under different sampling strategy was almost at the same level. In addition, the performance of PredictEFC on different folds was almost at the same level, suggesting that PredictEFC was quite stable for different folds.

**Table 5:** Performance of PredictEFC under tenfold cross-validation with stratified sampling

Fold	Absolute false	Absolute true	Aiming	Coverage	Accuracy
1	0.0490	0.8140	0.8388	0.8395	0.8313
2	**0.0383**	**0.8577**	**0.8776**	**0.8787**	**0.8706**
3	0.0478	0.8159	0.8494	0.8396	0.8354
4	0.0658	0.7531	0.7908	0.7835	0.7751
5	0.0504	0.8235	0.8393	0.8445	0.8351
6	0.0450	0.8235	0.8508	0.8655	0.8466
7	0.0450	0.8319	0.8571	0.8571	0.8487
8	0.0426	0.8445	0.8638	0.8676	0.8575
9	0.0496	0.8220	0.8326	0.8369	0.8305
10	0.0474	0.8213	0.8489	0.8496	0.8390
Mean	0.0481	0.8207	0.8449	0.8463	0.8370
Standard deviation	0.0072	0.0273	0.0231	0.0261	0.0252

### Comparison with the classifier using functional domain composition

Proteins’ functional domain information is widely used to investigate many protein-related problems. The traditional way to utilize such information for the protein representation is called functional domain composition. This section employed functional domain composition to represent enzymes, thereby building the classifier and comparing it with PredictEFC.

As mentioned in Sect. "Enzyme representation", 5117 IPR terms were involved for 2382 investigated enzyme. In this case, each enzyme was represented by a 5117-dimensional binary vector. These vectors, alone with the class labels of enzymes, were fed into RAKEL to construct classifiers. We also used RF and SVM with different kernel functions as base classification algorithms. The same parameters were tuned as mentioned in Sect. "Parameter selection". The best performance under different base classification algorithms is listed in Table 6. The range of accuracy was 0.7707–0.8545. Compared with the accuracies listed in Table 2, the accuracies of the classifiers using compact features varied in a small interval. However, the peak value of classifiers using functional domain composition was higher than those using compact features. The same phenomenon occurred for other four measurements. Among five classifiers using functional domain composition, the classifier with SVM (RBF kernel) yielded the best performance as it generated the highest performance on all five measurements. Accordingly, we selected this classifier to compare with PredictEFC.

**Table 6:** Performance of the multi-label classifiers using functional domain composition and different base classification algorithms

Base classification algorithm	Parameter	Absolute false	Absolute true	Aiming	Coverage	Accuracy	Time(s)
Random forest	*k* = 7, *I* = 500	0.0446	0.8350	0.8566	0.8484	0.8465	36,890.08
Support vector machine (Polynomial kernel)	*k* = 7, *C* = 1, *e* = 1	0.0434	0.8380	0.8610	0.8530	0.8505	**4956.18**
Support vector machine (Normalized polynomial kernel)	*k* = 5, *C* = 0.5, *e* = 2	0.0543	0.8018	0.8234	0.8162	0.8135	34,370.13
Support vector machine (Puk kernel)	*k* = 7, *C* = 5	0.0665	0.7607	0.7799	0.7721	0.7707	19,275.39
Support vector machine (RBF kernel)	*k* = 7, *C* = 7, *γ* = 0.1	**0.0423**	**0.8421**	**0.8650**	**0.8568**	**0.8545**	13,167.54

**Fig. 4:**
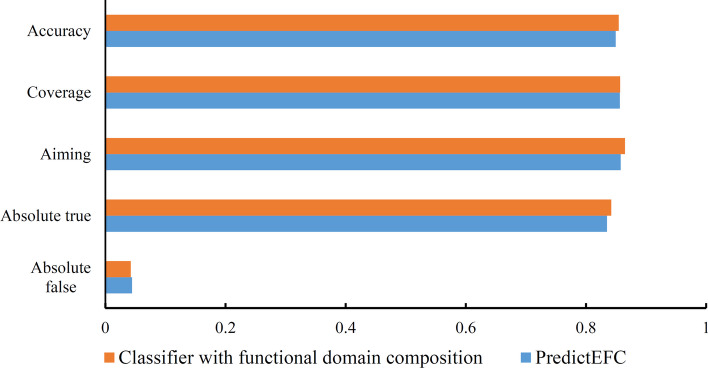
Bar chart to compare the overall performance of PredictEFC and the classifier with functional domain composition. These two classifiers give almost equal performance

**Fig. 5:**
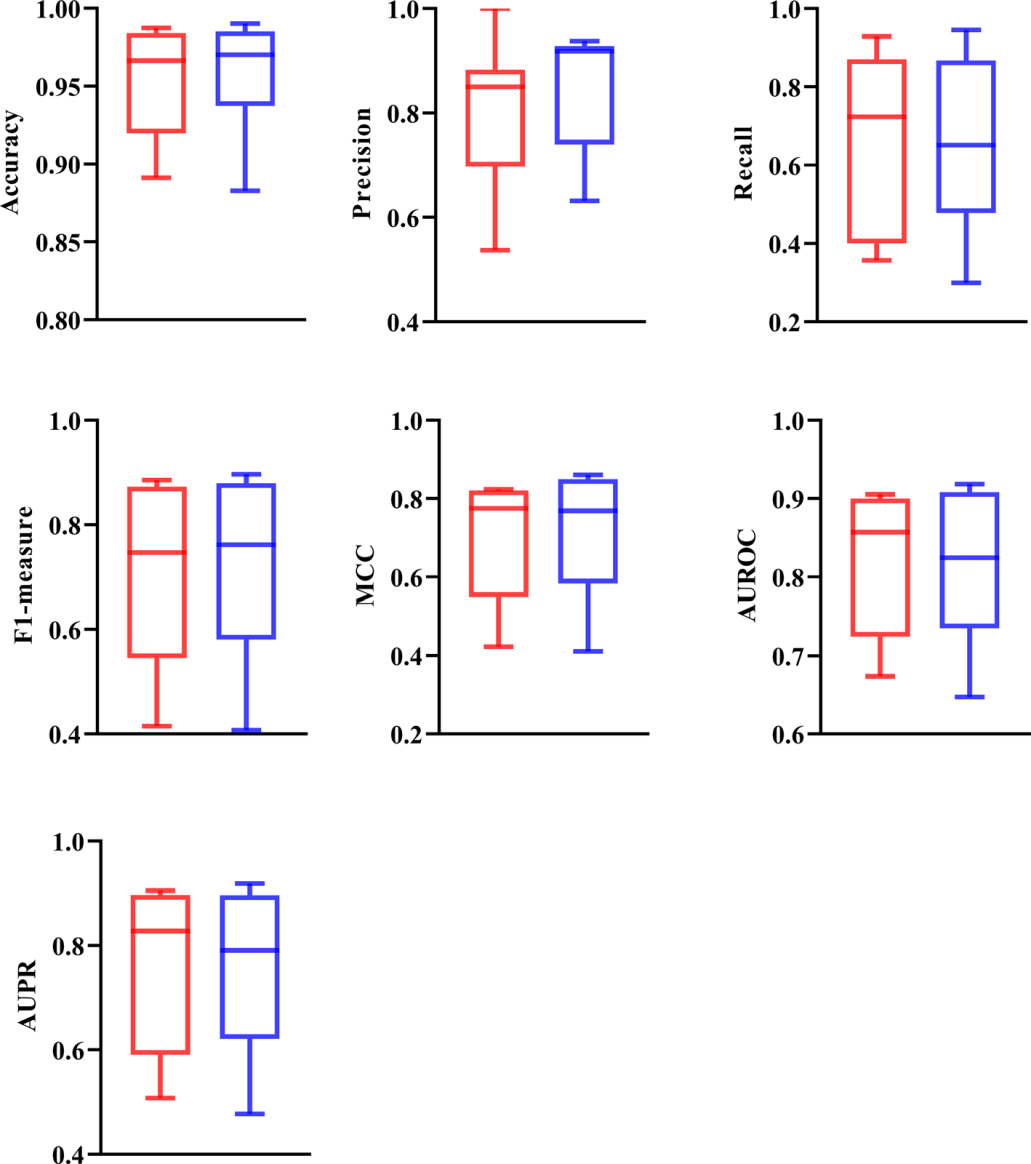
Box plot to compare performance of PredictEFC and the classifier with SVM (RBF kernel) and functional domain composition on seven family classes. The red and blue boxes represent the measurements of PredictEFC and the classifier with SVM (RBF kernel) and functional domain composition, respectively

For five overall measurements (Eqs. 9–13), PredictEFC produced a little lower performance on each measurement (see Fig. 4). In detail, the accuracy, absolute true, coverage and aiming were 0.0052, 0.0071, 0.0005, 0.0073, respectively, lower than those of the classifier using SVM (RBF kernel) and functional domain composition, whereas the absolute false was 0.0021 higher. Such gaps indicated that the performance of these two classifiers was almost at the same level. For other measurements representing the performance of classifiers on seven enzyme family classes, box plot was drawn for each measurement, as illustrated in Fig. 5. It can be observed that the ranges of all measurements except precision, including accuracy, recall, F1-measure, MCC, AUROC, and AUPR, were almost same for these two classifiers, further confirming their equal performance.

Besides the performance of the classifiers, the computing time is also an important indicator of the classifiers. Generally, the time follows an increasing trend on the dimension of the input vectors. In PredictEFC, enzymes were represented by 7-dimension vectors, whereas the classifier using functional domain composition adopted the 5117-dimension vectors. In theory, the efficiency of PredictEFC was much higher than that of the classifier using functional domain composition. To prove this fact, the cross-validation time was counted for classifiers using compact features or functional domain composition, as listed in the last column in Table 2 and 6. Evidently, the time for classifiers using compact features was much less than those using functional domain composition. The time for classifiers using compact features was less than a tenth of that of classifiers using functional domain composition.

With above arguments, the PredictEFC had similar performance but much higher efficiency to/than the traditional classifiers using functional domain composition. The novel feature extraction scheme based on functional domain information reserved the essential information of proteins and discarded useless information, improving the efficiency of classifiers but at the same time, the performance was not evidently reduced.

### Comparison with classifiers using traditional dimensionality reduction methods

The PredictEFC adopted the enzyme representation that was obtained by a deep analysis on enzymes’ functional domain information. The result can be deemed as the dimensionality reduction on functional domain composition. To indicate the superiority of the enzyme representation used in this study, some widely used dimensionality reduction methods were employed, which would be applied to the functional domain composition for obtaining the vectors of enzymes with lower dimensions. These methods included principal component analysis (PCA), singular value decomposition (SVD), non-negative matrix decomposition (NMF), t-distributed stochastic neighbor embedding (t-SNE), and uniform manifold approximation and projection (UMAP). Each above method was applied to the functional domain composition to obtain a new vector of each enzyme with a low dimension. For PCA, the various covered was set to different values (85%, 90% and 95%) to determine the dimension of vector. For SVD, the proportion of top singular values to all singular values (called singular value covered in this study) was employed to determine the dimension, which was set to 85%, 90% and 95%. As for NMF, the row vectors in the first matrix were picked up as the latent representations of enzymes. The dimension was set to various values between 100 and 1000. For t-SNE, perplexity was set to 30 and dimension was set to 2 and 3. For UMAP, number of neighbors was set to 15 and the dimension was set to various values between 5 and 20. Above dimensionality reduction methods have been implemented by corresponding packages in Scikit-learn [54], which were directly used in this study.

**Fig. 6:**
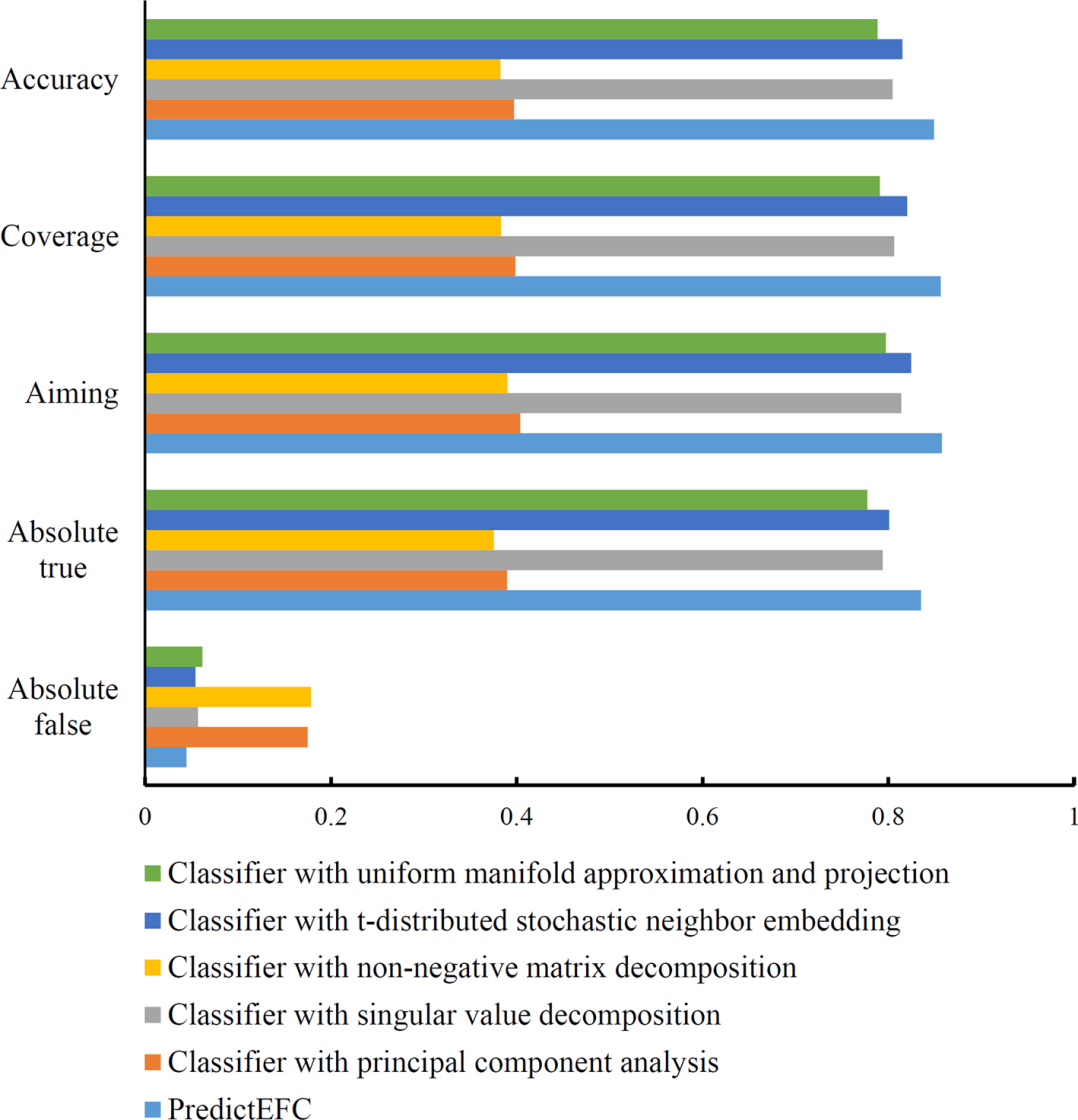
Bar chart to compare the overall performance of PredictEFC and the classifiers with traditional dimensionality reduction methods. PredictEFC is superior to other classifiers

Each new representation of enzymes with low dimensions was fed into RAKEL to set up the multi-label classifier, where the base classification algorithm was RF or SVM. Each classifier was also evaluated by tenfold cross-validation. The best performance, measured by accuracy, of each dimensionality reduction method is listed in Table 7. It can be observed that the accuracies for classifiers with PCA, SVD, NMF, t-SNE, and UMAP were 0.3970, 0.8046, 0.3825, 0.8152, and 0.7885, respectively. Compared with that of PredictEFC (Table 2), the accuracies of classifiers with PCA or NMF were much lower, the gap was more than 0.45; whereas the accuracy of the classifier with SVD, t-SNE, or UMAP was relatively close to that of PredictEFC, the gaps were between 0.03 and 0.07. Similar results occurred for other four measurements. For a clear display on the performance of above five classifiers and PredictEFC, a bar chart was plotted, as shown in Fig. 6. Evidently, PredictEFC provided better performance than other five classifiers. It was indicated that the novel scheme to reduce the dimension of functional domain composition was effective in retaining essential information of proteins.

**Table 7:** Performance of classifiers using functional domain composition processed by traditional dimensionality reduction methods

Dimensionality reduction method	Parameter	Absolute False	Absolute True	Aiming	Coverage	Accuracy	Time(s)
Principal component analysis	Various covered = 95%	0.1748	0.3896	0.4038	0.3984	0.3970	6054.56
Singular value decomposition	Singular value covered = 85%	0.0568	0.7939	0.8139	0.8064	0.8046	3023.14
Non-negative matrix decomposition	Dimension = 100	0.1784	0.3753	0.3899	0.3830	0.3825	3012.49
t-distributed stochastic neighbor embedding	Dimension = 2	**0.0542**	**0.8010**	**0.8248**	**0.8204**	**0.8152**	**315.50**
Uniform manifold approximation and projection	Dimension = 10	0.0615	0.7775	0.7974	0.7910	0.7885	602.84

On the other hand, we also counted the cross-validation time of the classifiers with PCA, SVD, NMF, t-SNE, and UMAP, which is listed in Table 7. The time for classifiers with PCA, SVD, and NMF exceeded 3000 s. Such time for PredictEFC was only 716.09 s, less than one fourth of the above time. This result suggested that PredictEFC had much higher efficiency than classifiers with PCA, SVD, and NMF. With the deep analysis on functional domain information of proteins, we can obtain a compact representation for enzymes and at the same time, the classifiers based on such representation had relative high performance. As for the cross-validation time for classifiers with t-SNE, it was much less than that of PredictEFC. The reason was that the feature dimension was only two. The cross-validation time of classifiers with UMAP was slightly less than that of PredictEFC. Considered the fact that above two classifiers provide lower performance than PredictEFC, PredictEFC was a more perfect classifier than these two classifiers to predict enzyme family classes.

### Performance of PredictEFC on two independent datasets

Two independent datasets were construct to test the generalization ability of PredictEFC, which were extracted from Expasy and KEGG ENZYME. The test results are listed in Table 8. On the independent dataset I, the PredictEFC yielded the absolute false of 0.0252, absolute true, aiming, coverage and accuracy of all 0.9118. This performance was even better than that on the benchmark dataset *S*, which are listed in Table 2. On the independent dataset II, the absolute false, absolute true, aiming, coverage, and accuracy of PredictEFC were 0.0349, 0.8705, 0.8777, 0.8849, and 0.8777. Likewise, this performance was also better than that on the benchmark dataset *S*. The comparison of performance of PredictEFC on two independent datasets implied that PredictEFC had better performance on the independent dataset I than independent dataset II. This result was reasonable because the independent dataset I was constructed from the same database (Expasy) to the benchmark dataset, whereas the independent dataset II was built from another database (KEGG ENZYME). Anyway, the performance of PredictEFC on two independent datasets was quite high, proving that PredictEFC had a strong generalization ability.

**Table 8:** Performance of different models on two independent datasets^a^

Model	Independent dataset	Absolute False	Absolute True	Aiming	Coverage	Accuracy
PredictEFC	Independent dataset I	**0.0252**	**0.9118**	**0.9118**	**0.9118**	**0.9118**
	Independent dataset II	0.0349	*0.8705*	*0.8777*	*0.8849*	*0.8777*
ECpred [22]	Independent dataset I	0.0588	0.6471	0.6471	0.6471	0.6471
	Independent dataset II	*0.0319*	0.8273	0.8273	0.8273	0.8273
BENZ WS [21]	Independent dataset I	0.0462	0.6765	0.6765	0.6765	0.6765
	Independent dataset II	0.0349	0.7842	0.7842	0.7842	0.7842

a: Bold numbers indicate the best performance on independent dataset I, whereas italic numbers denote the best performance on independent dataset II

### Comparison with previous models

Several computational models have been proposed to predict EC numbers of enzymes. As all previous models were established on different datasets, it was difficult to fairly compare PredictEFC with previous models. In view of this, we selected the models with web-server, including ECpred [22] and BENZ WS [21] for comparing their performance on two independent datasets. We directly input the sequences of enzymes in two independent datasets into above two web-servers and captured the predicted results for counting measurements listed in Eqs. 9–13. In another word, we tested the generalization ability of ECpred and BENZ WS. Obtained five measurements of these two models on two independent datasets are provided in Table 8. The performance of ECpred and BENZ WS on the independent dataset I was not very high. The accuracy and absolute true values were about 0.65. This performance was evidently lower than that of PredictEFC, which yielded accuracy and absolute true higher than 0.90. As for their performance on the independent dataset II, the absolute true and accuracy values were around 0.80, higher than those on the independent dataset I. However, they were still lower than those yielded by PredictEFC, which were about 0.87. Based on above results, it can be concluded that PredictEFC provided higher performance than ECpred and BENZ WS on two independent datasets, further proving the strong generalization ability of PredictEFC.

### Analysis of the effectiveness of the enzyme representation

In this study, we designed a novel scheme to count the distribution of each IPR term across seven family classes based on the enzymes in the training dataset, and this information was combined with the IPR terms of the given enzyme to generate the new representation of the given enzyme. Evidently, the distribution of IPR terms across seven family classes was a key factor to influence the quality of enzyme representation. This section gave an investigation on such information.

**Fig. 7:**
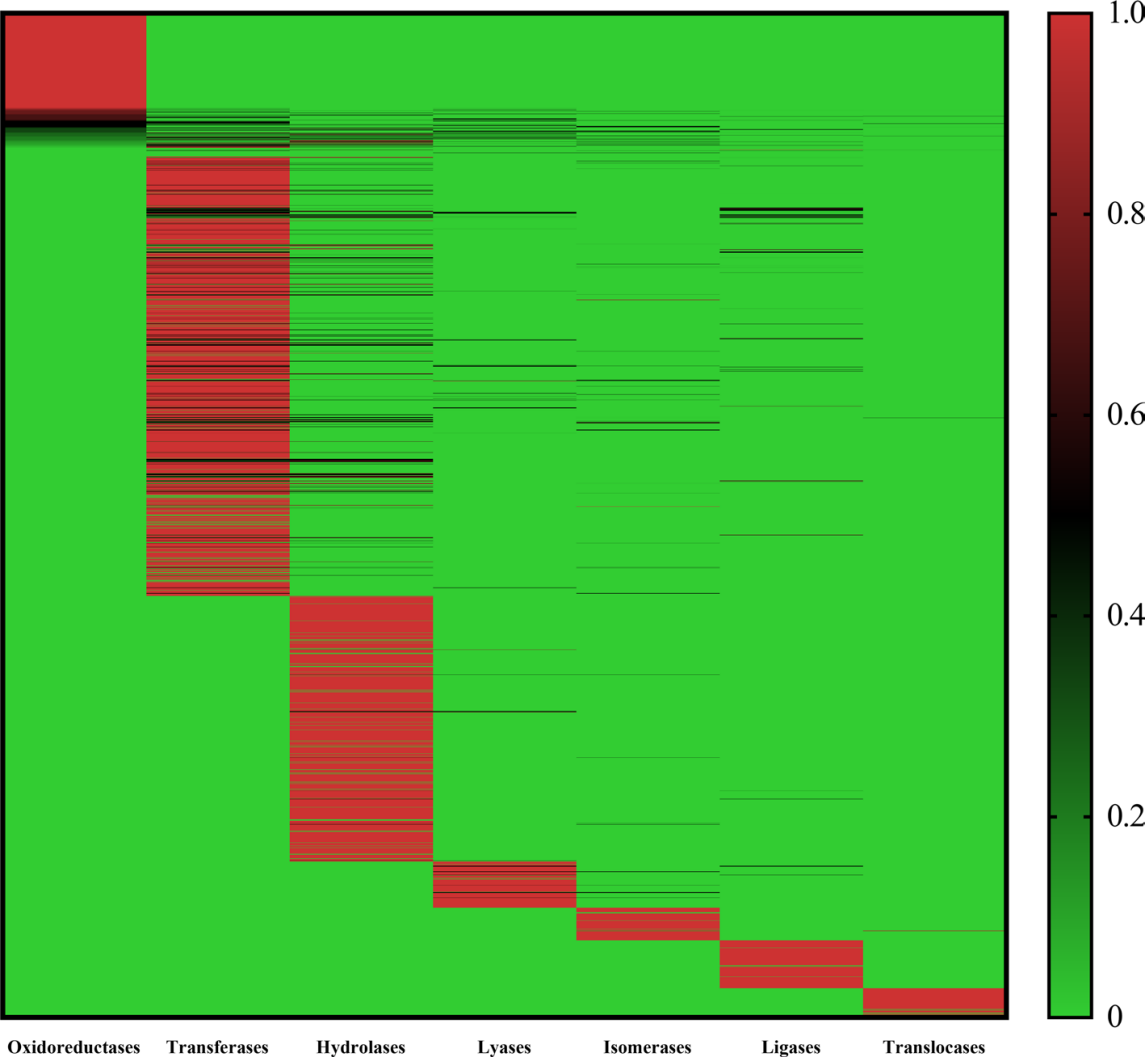
Heat map to show the distribution of IPR terms across seven family classes. Each family class has several exclusive IPR terms, meaning enzymes annotated by these IPR terms are more likely to be classified into the corresponding family class

**Fig. 8:**
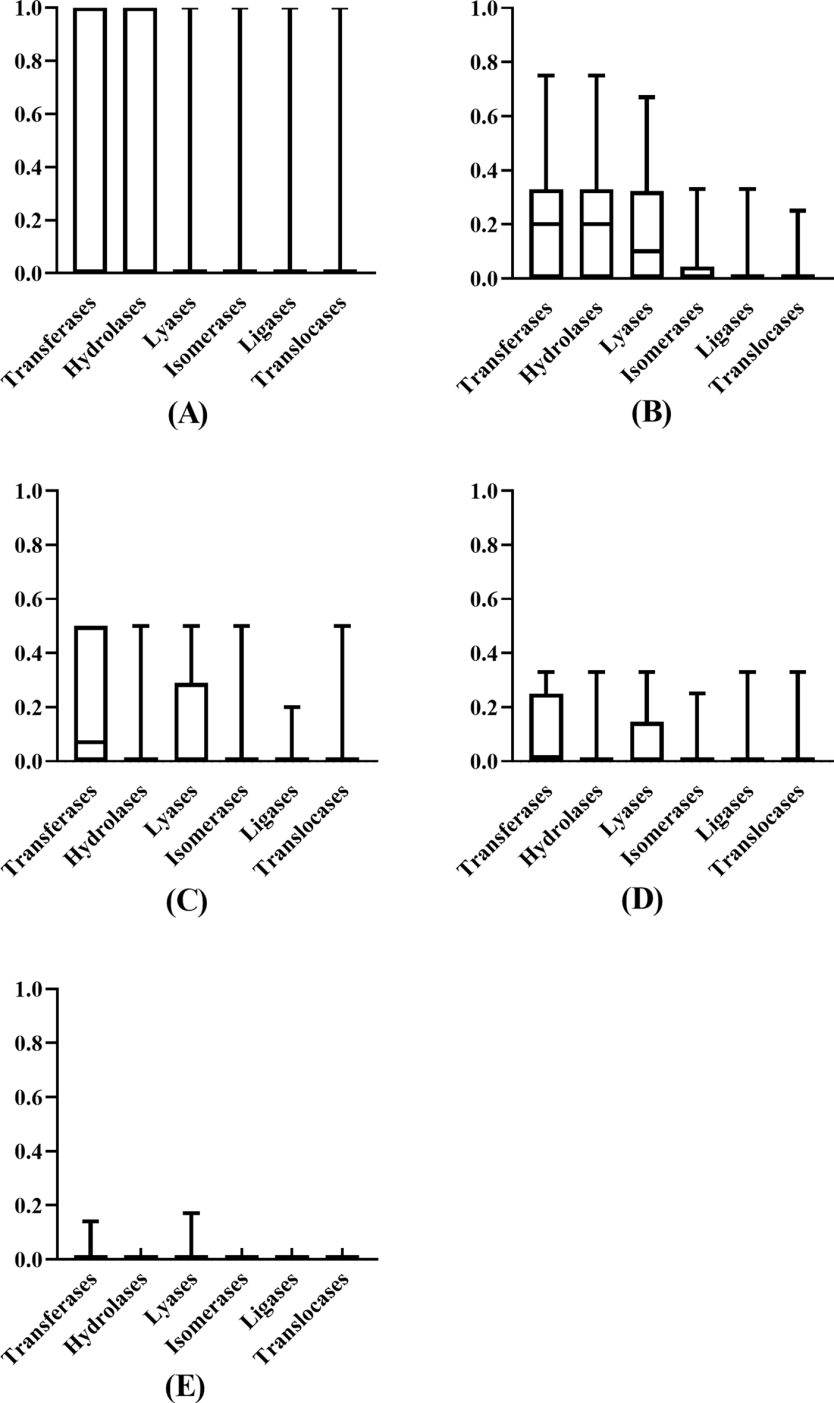
Box plot to show the distribution of IPR terms on six family classes according to the different ranges of their distribution on oxidoreductases. **A** the range is [0, 0.2]; **B** the range is (0.2, 0.4]; **C** the range is (0.4–0.6]; **D** the range is (0.6–0.8]; **E** the range is (0.8–1.0]

As mentioned in Sect. "Enzyme representation", we counted ${R}_{j}\left({IPR}^{i}\right)$ for the *i*-th IPR term and the *j*-th family class based on all 2382 enzymes. The results can be collected in a matrix with 5117 rows and 7 columns. A heat map was plotted for such matrix, as shown in Fig. 7. It can be observed that each family class has several exclusive IPR terms (the value of ${R}_{j}\left({IPR}^{i}\right)$ was close to one). Under such fact, the classifier is apt to classify the enzyme annotated by these IPR terms into the corresponding family class. Furthermore, for each family class, we first divided IPR terms into five groups according to their distributions on this family class, that is the IPR terms with distributions in [0, 0.2] constituted the first group, those with distributions in (0.2, 0.4], (0.4–0.6], (0.6–0.8] and (0.8–1.0] comprised the second, third, fourth and fifth groups, respectively. Under each group, the distributions of IPR terms in this group across other six family classes were counted and shown in box plot. The box plot for oxidoreductases is shown in Fig. 8 and those for other family classes are provided in Additional file 4. From Fig. 8, we can see that with the increasing of distributions on oxidoreductases, the distributions on other six family classes were generally reduced. Such phenomenon confirmed that IPR terms with high distributions on oxidoreductases were strongly related to this family class, whereas their linkages to other family classes were weak. The same conclusions can be obtained for other family classes (see Additional file 4). Above arguments suggested that the distributions of IPR terms across seven family classes had strong rules, which was very helpful to extract informative features, thereby building efficient classifiers.

### Analysis of PredictEFC on domain frequency

**Fig. 9:**
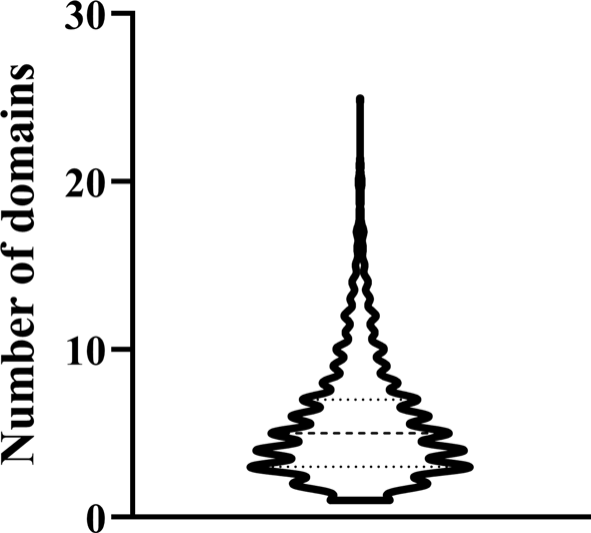
Violin plot to show the distribution of numbers of domains annotated to enzymes in the benchmark dataset. Most enzymes are annotated to less than 10 domains

There were 2382 enzymes in the benchmark dataset *S*. The numbers of domains annotated to different enzymes were remarkably changed. A violin plot shows the distributions of numbers of domains annotated to 2382 enzymes, as illustrated in Fig. 9. It can be observed that most enzymes were annotated by less than 10 domains, some enzymes were annotated by only one domain, whereas the enzymes “P49327” and “P27708” had the most domains (25). As the proposed model, PredictEFC, was constructed based on domains of enzymes. It was interesting to investigate the influence of domain frequency on PredictEFC. To this end, we divided 2382 enzymes into three groups. The first group contained 100 enzymes with most domains, the second group consisted of 100 enzymes with least domains, and the rest enzymes comprised the last group. For the predicted results yielded by the general tenfold cross-validation, the five measurements on above three enzyme groups were counted individually, which are provided in Table 9. It can be found that the performance of PredictEFC was highly related to the domain frequency. For enzymes annotated by few domains, the performance of PredictEFC was not very high. The accuracy was only 0.7400. Its performance increased with the increasing in domain frequency. The accuracy for enzymes annotated by middle domains raised to 0.8422, and that for enzymes annotated by most domains achieved maximum of 0.9042. Based on the above results, the predicted family classes of enzymes annotated by many domains were generally more reliable than those of enzymes annotated by few domains. This result was reasonable because the quantity of domains determined the abundance of features. Few domains provided limited essential information of enzymes, whereas many domains gave the abundant core information of enzymes.

**Table 9:** Performance of PredictEFC on enzymes annotated by few, middle and many domains

Quantity of domains	Absolute False	Absolute True	Aiming	Coverage	Accuracy
Few	0.0757	0.7400	0.7400	0.7400	0.7400
Middle	0.0462	0.8272	0.8506	0.8499	0.8422
Many	**0.0300**	**0.8700**	**0.9250**	**0.9192**	**0.9042**

### Performance of the PredictEFC on Yeast

So far PredictEFC was only tested on human enzymes. This section further tested its performance on enzymes of another specie, Yeast. The Yeast enzymes were also retrieved from Expasy (accessed in August 2022), resulting in 1449 enzymes. With the same data cleaning procedures for human enzymes, 1165 Yeast enzymes were obtained for building and testing PredictEFC. Their distribution on seven enzyme family classes is provided in Table 10. It can be observed that this enzyme dataset was smaller than the benchmark dataset *S*, however, the MD was almost same. We still used the same scheme to encode Yeast enzymes (see Sect. "Enzyme representation") and RAKEL with SVM or RF as base classification algorithm to set up the classifier. The grid search was also applied to the parameter values mentioned in Sect. "Parameter selection" to extract optimal parameters. According to the tenfold cross-validation results of classifiers with all possible parameter combinations, the final classifier for Yeast selected SVM with polynomial kernel as the base classification algorithm, *C*, *k*, and *e* were set to 3, 7, and 1, respectively. For convenience, this classifier was also called PredictEFC. Its performance under general tenfold cross-validation is listed in Table 11. The five measurements were 0.0633, 0.7702, 0.7923, 0.7862, and 0.7826. Table 11 also lists its performance under the tenfold cross-validation with stratified sampling, indicating absolute false of 0.0672, absolute true of 0.7559, aiming of 0.7792, coverage of 0.7742, and accuracy of 0.7695. Same to the results on human enzymes, the performance under tenfold cross-validation with stratified sampling was lower. However, the difference was very small. Compared to the cross-validation results on human enzymes, the performance of PredictEFC on Yeast enzymes decreased. However, the decrease is not very remarkable. PredictEFC still provided high performance on Yeast enzymes. It is believed that PredictEFC can be transplanted for predicting enzyme family classes of other species.

**Table 10:** Breakdown of the Yeast enzymes

Tag	Enzyme family class	Number of enzymes
EC 1	Oxidoreductases	158
EC 2	Transferases	493
EC 3	Hydrolases	327
EC 4	Lyases	75
EC 5	Isomerases	48
EC 6	Ligases	75
EC 7	Translocases	21
Sum		1197
Number of different enzymes		1165
The multiplicity degree MD		1.027

**Table 11:** Performance of PredictEFC on Yeast enzymes

Cross-validation	Absolute false	Absolute true	Aiming	Coverage	Accuracy
General tenfold cross-validation	**0.0633**	**0.7702**	**0.7923**	**0.7862**	**0.7826**
tenfold cross-validation with stratified sampling	0.0672	0.7559	0.7792	0.7742	0.7695

### Web-server and user guide

**Fig. 10:**
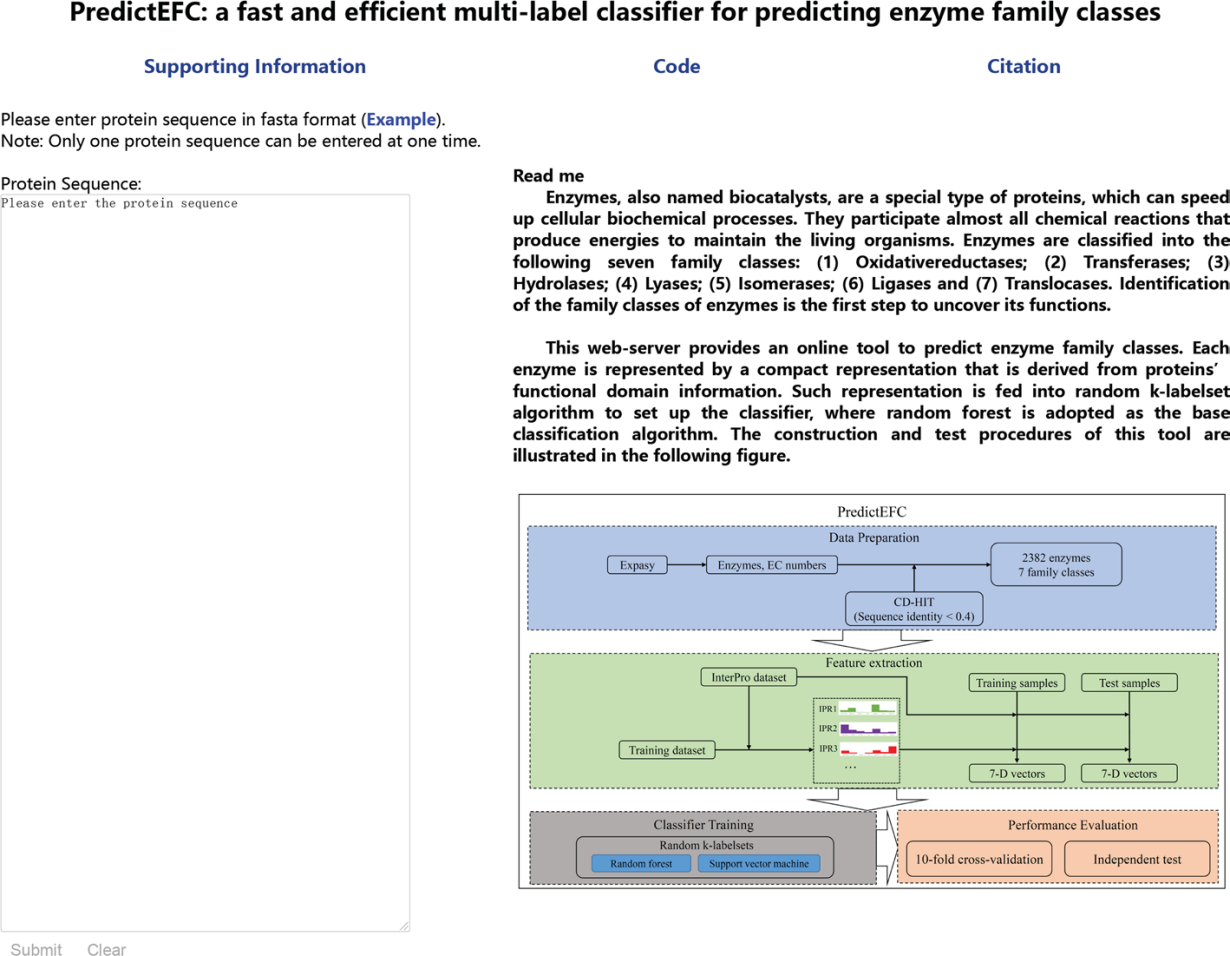
Home page of the web-server

For easy usage of PredictEFC, a web-server with the same name was developed, which can be accessed at http://124.221.158.221/. The home page is illustrated in Fig. 10. There are three tabs at the top of home page, including Supporting Information, Code and Citation. In the tab of Supporting Information, two datasets are provided: (1) labels of 2382 enzymes; (2) features of 2382 enzymes. It is necessary to point out that the features of 2382 enzymes are for training the final classifiers, which are different from those used for tenfold cross-validation. In the tab of Code, codes for this web-server are provided, along with the supporting materials. In the tab of Citation, the reference for this web-server is available. In the right of home page, a brief description of this web-server is given. In the left of home page, a text box is placed for receiving input. Users can use the following steps to submit their input and access the results.

Step1. Users input the protein sequence with fasta format in the text box in the left of home page and click Submit button to submit the sequence to the web-server. If users input a wrong sequence, they can use Clear button to remove the original input and give a new input.

Step2. After submitting the sequence, users can access the results within 2–3 min. The result page displays the name of seven family classes and the predicted classes of the input sequence.

Step3. Users can click Back button to return the home page.

### Limitations of this study

This study proposed an efficient classifier for predicting enzyme family classes. Although this classifier had some merits, it still had some limitations. First, only two base classification algorithms (RF and SVM) were attempted when constructing the classifier. It was not clear whether this selection was optimal. Employment of other classification algorithms may yield a more powerful classifier. Second, the classifier was built using traditional machine learning algorithms, which restricted its performance. The newly designed deep learning algorithms provided abundant resources for designing excellent classifiers. Third, the proposed classifier strongly relied on the functional domain information of enzymes. For the enzymes annotated by no domains or few domains, the classifier cannot provide reliable results. Finally, this study only focused on enzyme family classes (the first EC number), causing the proposed classifier cannot recognize non-enzyme proteins and EC numbers at high levels. This limited the applications of PredictEFC. In future, we will continue this work to set up more perfect classifiers.

## Conclusions

This study proposed a new multi-label classifier for predicting family classes of enzymes. In this classifier, each enzyme was represented by a compact vector containing seven components, which was yielded by a novel feature extract scheme designed for processing functional domain information. The experiment results indicated that the classifier had good performance as well as high efficiency. The classifier was competitive for classifiers using traditional schemes and previous classifiers, and the running time was sharply reduced. The user-friendly web-sever was also set up, which was easy to use for any users without computer science background. It is hopeful that the newly proposed classifier can be a useful tool for the large-scale test on candidate enzymes and the newly proposed feature extraction scheme on functional domain information can be applied to deal with other protein-related problems.

### Supplementary Information

**Additional file 1**. Benchmark dataset retrieved from Expasy

**Additional file 2**. Independent dataset retrieved from Expasy

**Additional file 3**. Independent dataset retrieved from KEGG ENZYME

**Additional file 4**. Box plot to show the distribution of IPR terms on six family classes according to the different ranges of their distribution on a given family class

## Data Availability

All data analysed during this study are included in this published article and its supplementary information files. The source codes are available at http://124.221.158.221/.

## Abbreviations

EC: Enzyme commission
ANN: Artificial neuron network
SVM: Support vector machine
NNA: Nearest neighbor algorithm
LDA: Linear discriminant analysis
RAKEL: Random k-labelsets
RF: Random forest
MD: Multiplicity degree
LP: Label powerset
TP: True positive
FP: False positive
TN: True negative
FN: False negative
PCA: Principal component analysis
SVD: Singular value decomposition
NMF: Non-negative matrix decomposition
t-SNE: T-distributed stochastic neighbor embedding
UMAP: Uniform manifold approximation and projection
