# Supplementary material for: PredictEFC: a fast and efficient multi-label classifier for predicting enzyme family classes
Source: BMC Bioinformatics. 2024 Jan 30;25:50. doi: 10.1186/s12859-024-05665-1 (PMC10829269; doi:10.1186/s12859-024-05665-1)
Supplement: Supplementary file 1 — Additional file 1. Benchmark dataset retrieved from Expasy [file 12859_2024_5665_MOESM1_ESM.docx]

**Additional file 1.** Benchmark dataset retrieved from Expasy.

**1. Oxidoreductases**

| P40394 | Q00796 | Q8N335 | Q7Z4W1 | O60701 | Q9BYZ2 | Q9BUT1 | Q02338 |
| --- | --- | --- | --- | --- | --- | --- | --- |
| P31937 | P04035 | Q08426 | Q99714 | Q16836 | P40925 | P40926 | O43175 |
| Q16798 | P50213 | P48735 | P52209 | Q9Y2S2 | O95479 | P11413 | P14061 |
| Q9BPW9 | Q53GQ0 | Q9BPX1 | P37059 | P56937 | Q92506 | Q6UWP2 | P16152 |
| Q9UBQ7 | Q8N4T8 | P49327 | Q06136 | A6NNS2 | Q8N3Y7 | P15428 | P14060 |
| P28845 | P35270 | Q15738 | Q9UQ10 | Q9H2F3 | Q9BTZ2 | P20839 | P40939 |
| Q13630 | O75911 | Q8IZV5 | Q9HBH5 | O43488 | P51659 | Q86WU2 | Q9UJM8 |
| P43304 | Q8NE62 | Q9H9P8 | Q8IWW8 | Q8N465 | P51648 | P49419 | P49189 |
| O14556 | Q02252 | P51649 | Q9H2A2 | P54886 | Q8WVX9 | P30038 | Q06278 |
| P08559 | P11177 | Q02218 | P12694 | P21953 | Q12882 | P51857 | Q9UBM7 |
| Q9H8P0 | P18405 | P53004 | P30043 | Q9BY49 | Q14914 | Q8N8N7 | Q8N4Q0 |
| Q14739 | Q15392 | Q6P1R4 | Q96G46 | O95620 | Q9NX74 | Q9NZ01 | Q9BV79 |
| Q9NUI1 | Q16698 | P36551 | P50336 | O15254 | P31040 | P21912 | Q02127 |
| P16219 | P26440 | P45954 | Q92947 | P11310 | P28330 | P49748 | Q6NUM9 |
| P00367 | Q96RQ9 | P14920 | P21397 | Q9NVS9 | Q9Y4K0 | P28300 | Q16853 |
| P19801 | P23378 | Q96C36 | Q86XF0 | Q3SY69 | Q9UDR5 | P13995 | P42898 |
| Q14894 | P09417 | Q9P0Z9 | Q6QHF9 | Q9NWM0 | Q16134 | Q9UF12 | O43272 |
| Q9UL12 | Q9UI17 | Q9UHQ9 | Q7L1T6 | Q6IPT4 | P16435 | Q9NRD9 | Q8TDZ2 |
| Q5VYX0 | P15559 | Q53FA7 | Q08257 | Q9P2T1 | P09622 | P00390 | Q6DKJ4 |
| P62341 | Q16881 | P51687 | P55789 | O00391 | Q6ZRP7 | Q13228 | Q9UHG3 |
| Q8NBK3 | O95881 | Q9UJ68 | Q9NZV6 | Q8IXL7 | Q9H4Y5 | Q9Y6N5 | Q9BYN0 |
| P04040 | P07202 | P22352 | Q8TED1 | P36969 | Q8TBF2 | Q13162 | P30044 |
| P30041 | Q93099 | P46952 | P48775 | O95571 | Q96SZ5 | Q16878 | O00625 |
| P32754 | P09917 | P14902 | Q9BV57 | Q9HAY6 | Q9BYV7 | Q9UGB7 | O75936 |
| O15460 | Q7Z4N8 | O60568 | Q32P28 | Q9NVH6 | Q12797 | O14832 | Q8NHM5 |
| Q9UPP1 | Q9H6W3 | Q9GZT9 | Q9NXG6 | Q9NWT6 | Q13686 | Q6NS38 | Q96Q83 |
| A2RUC4 | Q9NXW9 | Q6P6C2 | Q9C0B1 | P0C870 | O43593 | Q9Y4C1 | Q7LBC6 |
| Q6ZMT4 | O94953 | Q9H3R0 | P29375 | O15550 | O15054 | Q8N371 | Q8NFU7 |
| Q6N021 | O43151 | P31512 | O15229 | P29475 | P35228 | O94851 | Q7RTP6 |
| P05177 | Q16678 | Q9HB55 | Q86W10 | P11511 | P08686 | Q14534 | P30519 |
| P05093 | Q9Y6A2 | Q9NYL5 | O75881 | P51589 | Q6ZWL3 | Q7Z449 | Q9UNU6 |
| Q16850 | Q6NT55 | P15538 | P05108 | Q02318 | Q07973 | P07101 | Q6ZNB7 |
| P09172 | P19021 | P14679 | Q6QHC5 | Q15800 | O00767 | O95864 | O75845 |
| Q4G0S4 | A5PLL7 | P35354 | Q9BU89 | O95992 | Q99807 | O60341 | Q8NB78 |
| P08294 | P04179 | Q9Y4U1 | Q9UBK8 | Q687X5 | Q16595 | Q8N4E7 | Q6MZM0 |
| P23921 | P31350 | Q8N0U8 | Q99424 | P22570 | Q6PHW0 | P55073 | Q92813 |

**2. Transferases**

| P49327 | P54886 | Q9H4Y5 | P30041 | O60568 | Q9Y4U1 | P40261 | Q14353 |
| --- | --- | --- | --- | --- | --- | --- | --- |
| Q93088 | P21964 | Q8WZ04 | P50135 | Q6UX53 | Q99707 | Q9UBM1 | Q14749 |
| Q8N4J0 | P11086 | Q9UBP6 | Q8IZ69 | Q96GJ1 | Q9Y6K1 | P26358 | P04818 |
| O43148 | Q8N1G2 | Q7Z624 | Q9H4Z3 | P16455 | Q9NZJ6 | P51580 | Q8N3J2 |
| P22061 | O95568 | Q86TU7 | O60725 | Q9HBK9 | Q9UNQ2 | Q5HYK3 | Q08J23 |
| O14717 | Q9UET6 | Q8IYL2 | Q9NXH9 | Q7L0Y3 | Q9BVS5 | Q96FX7 | Q8TBZ6 |
| Q6PF06 | Q9NUP7 | Q32P41 | Q96BT7 | Q9UIC8 | Q9BV44 | Q6IPR3 | O60294 |
| Q8IYT2 | Q9Y5R4 | Q5VVY1 | Q9H2P9 | P55345 | O60678 | Q96LA8 | Q86X55 |
| Q86XA0 | O14744 | Q6P2P2 | Q7L592 | Q9NVM4 | Q86W50 | Q86U44 | Q9NQV7 |
| Q8NB12 | Q9NRG4 | Q9H7B4 | Q13029 | O43463 | Q92800 | Q96L73 | Q53H47 |
| Q9NR48 | Q9BYW2 | Q9C0A6 | Q8TEK3 | Q9NQR1 | Q4FZB7 | Q86Y97 | Q9NQX0 |
| Q03164 | Q9UMN6 | Q8NEZ4 | O14686 | O15047 | Q9UPS6 | Q9NVD3 | Q8WTS6 |
| Q15047 | Q96T68 | Q9H9B1 | Q03112 | Q9HAZ2 | Q9BZ95 | Q6GMV2 | Q5T8I9 |
| P34897 | P22102 | P31939 | O95954 | Q96DP5 | P48728 | P27708 | P00480 |
| P50440 | Q9H0I9 | P37837 | Q8N159 | Q96EK6 | P18440 | P28329 | P09110 |
| P24752 | P10515 | Q6IB77 | Q5SZD4 | Q9HCL2 | Q6NUI2 | Q86UL3 | P55084 |
| P22307 | Q8N9F0 | O75907 | Q96PD7 | Q8TCG5 | P23786 | Q92604 | Q6ZWT7 |
| Q6P1A2 | Q7L5N7 | Q9HA82 | P35610 | O75600 | P13196 | Q8IVS2 | Q9NWU1 |
| O15228 | P04180 | Q96PB1 | Q92793 | Q9Y6F7 | O15516 | O60318 | O14929 |
| Q92830 | Q92993 | Q92794 | Q8WYB5 | O95251 | Q9H7X0 | Q15788 | Q9Y6Q9 |
| P21675 | Q00403 | Q9UKN8 | O15269 | O15270 | Q8WTS1 | Q6UWP7 | Q99943 |
| Q9NRZ5 | Q9NUQ2 | Q9NST1 | P21673 | P36957 | Q969I3 | Q68CP4 | Q9UHE5 |
| Q16613 | O60551 | Q5SQI0 | O95237 | Q9UKG9 | P11182 | A6NK58 | Q9HB03 |
| Q9GZR5 | P15104 | Q9H8X9 | Q96GR4 | Q8IZN3 | Q969W1 | Q8IUH5 | Q8WVZ1 |
| Q8IVQ6 | Q8N966 | Q8IYP9 | Q6UX98 | Q8WTX9 | Q9UIJ5 | Q9NPG8 | Q9H6R6 |
| Q9NXF8 | Q9ULC8 | Q9NPF4 | Q9H4B0 | Q9H237 | P61599 | P41227 | Q147X3 |
| Q86UY6 | Q9GZZ1 | Q96EB6 | Q9NTG7 | Q9Y6E7 | Q8N6T7 | Q9NRC8 | Q8N8W4 |
| P27544 | A6NGU5 | P36269 | Q6P531 | Q9UJ14 | Q9NXS2 | O95260 | P22735 |
| Q08188 | P49221 | O43548 | Q969T4 | Q9Y385 | Q8N2K1 | P68036 | Q7Z7E8 |
| Q712K3 | P49459 | O00762 | P62256 | P61086 | Q16763 | Q9NPD8 | Q5VVX9 |
| Q96B02 | Q9H832 | Q9C0C9 | O15033 | Q7L622 | Q8IYU2 | Q9ULT8 | Q5U5R9 |
| Q5T447 | Q9Y4D8 | Q76N89 | Q15751 | O95714 | Q5GLZ8 | Q8IVU3 | Q7Z6Z7 |
| P46934 | Q9HCE7 | Q14669 | Q05086 | Q7Z3V4 | Q15386 | Q7Z6J8 | O95071 |
| Q9H0M0 | Q99728 | Q13490 | Q9NR09 | Q96CA5 | Q7Z569 | P38398 | O75150 |
| Q13191 | Q96EP1 | Q9UNE7 | O43734 | Q9NPC3 | O95628 | Q8NHY2 | Q5T197 |
| Q86UW9 | Q8TDB6 | Q8N9I9 | Q86Y13 | Q66K89 | Q9NW38 | Q86XS8 | Q75N03 |
| Q14527 | Q8IU81 | Q9H1B7 | Q9NQC1 | Q9P0J7 | Q2Q1W2 | Q8TBB1 | Q6UWE0 |
| O94822 | Q7L5Y9 | Q86UD3 | Q9P2E8 | Q9NX47 | O60337 | Q9H992 | Q5T0T0 |
| Q8NA82 | Q00987 | Q5U5Q3 | Q86YT6 | Q96AX9 | Q9H000 | Q13064 | Q969V5 |
| Q8WY64 | O76050 | Q96EH8 | Q6VVB1 | Q9Y314 | Q8WV22 | Q5W0B1 | Q8N2H9 |
| Q8N2W9 | O43164 | O60927 | Q13356 | Q9UMS4 | Q9UPQ7 | O15541 | Q9NS91 |
| P15918 | Q7Z6E9 | P62877 | Q9HBD1 | Q6PCD5 | Q06587 | Q96G75 | O00237 |
| Q6ZNA4 | Q9ULX5 | Q5XPI4 | Q96EQ8 | Q9BV68 | Q8TEB7 | Q8IUD6 | Q8WVD3 |
| Q8WU17 | Q96MT1 | Q9NTX7 | Q8N8N0 | Q96PX1 | Q96A37 | Q8IYW5 | Q8NCN4 |
| Q96K19 | Q86T96 | Q9P0P0 | Q8N6D2 | Q96D59 | Q96GF1 | Q9NXI6 | Q5TA31 |
| Q63HN8 | Q9NWF9 | Q5VTB9 | Q9NVW2 | O43567 | Q96BH1 | Q9BY78 | Q969K3 |
| O94941 | Q9H0F5 | Q9H4P4 | Q68DV7 | P78317 | Q9Y252 | O76064 | Q5M7Z0 |
| Q7Z6J0 | Q8TEC5 | Q8TEJ3 | Q149N8 | O43255 | Q86TM6 | Q13263 | A0AVI4 |
| Q9NS56 | Q12933 | Q13114 | Q9BUZ4 | Q9Y4K3 | Q6Q0C0 | Q9BWF2 | O60858 |
| O95361 | Q9Y577 | Q8IYM9 | P36406 | Q14258 | Q12899 | P14373 | Q9BZY9 |
| Q13049 | Q9UPN9 | Q9UPQ4 | Q9NQ86 | O94972 | Q9HCM9 | Q6P9F5 | Q8WV44 |
| Q96LD4 | Q8IWZ4 | Q86XT4 | Q9BRZ2 | Q8NG06 | Q9BVG3 | Q969Q1 | Q86WT6 |
| Q9UJV3 | Q9C040 | Q9C037 | Q9C029 | Q9BZR9 | Q9C026 | Q8N9V2 | Q8N7C3 |
| P53804 | Q14139 | O95155 | Q8IWV8 | Q6ZT12 | Q5T4S7 | Q8N806 | Q96PU4 |
| P98170 | Q96JP5 | Q96PM5 | Q86UK7 | Q8NHG8 | Q9ULT6 | Q8WWF5 | Q8NEG5 |
| Q9P2G1 | Q9Y4X5 | O95376 | Q9BYM8 | O60260 | Q7Z419 | Q9NV58 | Q8TC41 |
| Q9UBS8 | Q96EP0 | Q969M7 | O75592 | P61081 | Q9UKV5 | O75390 | P53396 |
| Q8N0X4 | Q01581 | P06737 | P13807 | P0DTE5 | Q6NUS8 | Q04446 | P15291 |
| P35573 | P16442 | Q10471 | Q14435 | Q7Z7M9 | Q86SF2 | Q9NY28 | Q86SR1 |
| Q8NCW6 | Q8N3T1 | Q16880 | Q8IYK4 | O96024 | Q9BYC5 | P19526 | O75752 |
| Q16739 | O60762 | O43825 | Q00973 | P26572 | Q9P109 | Q9Y6A1 | Q9UKY4 |
| Q8IUR5 | Q8N394 | Q6ZXV5 | Q5T4D3 | Q9Y673 | Q9NS00 | Q2TAA5 | Q9H553 |
| Q9UBV7 | Q96L58 | O94766 | Q9NP73 | Q9BT22 | Q10469 | Q09327 | Q9UQ53 |
| Q9UBM8 | Q6ZMB0 | Q9NY97 | Q9C0J1 | Q8N0V5 | P22083 | Q9Y231 | Q3V5L5 |
| Q8N6G5 | Q8IZ52 | Q70JA7 | O15488 | P37287 | Q9BYG0 | Q92839 | Q9H488 |
| Q9Y2G5 | Q8NES3 | Q9UBQ6 | O43909 | Q16394 | Q93063 | Q92935 | Q9NPC4 |
| Q6L9W6 | Q76KP1 | Q5NDL2 | O15294 | Q5BKT4 | Q92685 | Q9H6U8 | Q9BV10 |
| Q9BVK2 | Q9Y672 | O43286 | Q8NAT1 | Q8NCR0 | Q8NBL1 | Q8IV20 | P00491 |
| O95045 | P19971 | P07741 | P00492 | P11172 | P43490 | Q06203 | Q15274 |
| Q86Y38 | Q13126 | P09874 | Q9UGN5 | O95271 | P52961 | Q13508 | Q93070 |
| Q96L15 | A0PJZ3 | Q8NBI6 | Q9BXR0 | Q96JF0 | Q16842 | Q11206 | Q9NSC7 |
| Q9UJ37 | Q11203 | Q8NDV1 | Q92185 | Q9UNP4 | Q8TCJ2 | P28907 | P14324 |
| O95749 | Q00266 | P19623 | P0CG29 | P08263 | Q9Y2Q3 | P21266 | P09211 |
| O60760 | O43813 | O43708 | P10620 | O14684 | P37268 | P52788 | Q8N5C7 |
| Q8NBA8 | O00116 | Q96H96 | P49366 | Q9NR45 | P49354 | P49356 | P53609 |
| Q92696 | P53611 | P08397 | Q9H3H1 | Q86SQ9 | Q96E22 | Q86YH6 | Q5T2R2 |
| Q9BZG8 | Q53H54 | Q12887 | Q8NHS2 | P00505 | Q8TD30 | Q8N5Z0 | P17735 |
| Q6YP21 | P04181 | Q06210 | P80404 | Q9BYV1 | O15382 | P21549 | Q9Y617 |
| P52790 | P50053 | P51570 | Q01813 | Q5T6J7 | Q9UHJ6 | Q9H477 | O75191 |
| P55263 | P04183 | O00142 | Q9NPI5 | O95544 | Q4G0N4 | Q13057 | O43252 |
| Q969G6 | Q3LXA3 | Q6ZS86 | P32189 | Q8IVS8 | P35790 | Q8TE04 | O00764 |
| Q03426 | P30613 | Q96C11 | Q9NWZ5 | Q8N0W3 | Q9UJ70 | Q9Y223 | Q8TCG2 |
| P42356 | Q9UBF8 | O60331 | Q5T9C9 | Q16854 | P49902 | Q92989 | Q96T60 |
| A2RU49 | Q9HBU6 | Q9NRA0 | Q53H12 | Q16875 | Q6PCE3 | Q9Y6T7 | P52429 |
| Q86XP1 | O75912 | Q5KSL6 | P52824 | Q9UPQ8 | P27987 | Q96DU7 | Q13572 |
| O00443 | O75747 | Q8NEB9 | P42336 | P48736 | Q8TCT0 | Q8NFU5 | Q9BRR6 |
| Q8TBX8 | Q9Y2I7 | P42338 | Q01415 | Q9H8X2 | Q86TN4 | Q8IV42 | Q9H479 |
| Q9H5K3 | P00558 | P17540 | Q15126 | P54819 | Q9Y6K8 | Q9Y3D8 | Q96M32 |
| Q96MA6 | Q5TCS8 | Q5EBM0 | P27144 | P30085 | O75414 | Q9Y5B8 | O00746 |
| Q16774 | P23919 | Q92551 | Q6PFW1 | P60891 | Q9H3S4 | Q9HAN9 | Q9BZQ4 |
| Q8NFF5 | O95602 | Q9H9Y6 | P24928 | P30876 | O14802 | Q9NW08 | O00411 |
| P28340 | Q07864 | P54098 | P09884 | P06746 | Q9UGP5 | Q7Z5Q5 | O75417 |
| P63128 | Q9Y253 | Q9UNA4 | Q9UBT6 | O60673 | Q8TCS8 | Q16851 | P07902 |
| Q9Y5P6 | Q99447 | Q9Y5K3 | Q6PIY7 | Q9NVV4 | Q5XG87 | P51003 | Q9H6E5 |
| Q96IP4 | Q16222 | O14772 | P04053 | A4D126 | Q92903 | Q96BW9 | Q8NFW8 |
| O00370 | P63135 | O14746 | O60942 | P49789 | Q5TAX3 | Q5VYS8 | Q96Q11 |
| Q9NQX3 | Q6ZNW5 | Q9NWX6 | O95396 | Q9Y6K5 | Q8N884 | Q86U90 | Q9UKK9 |
| P49642 | Q9BVA6 | Q9BVL4 | Q9Y6K0 | Q9C0D9 | Q32NB8 | Q9NRN7 | O14735 |
| Q9H3H5 | Q3T906 | Q86VZ5 | P48651 | Q9BVG9 | Q9UJA2 | Q99611 | Q9UM73 |
| Q08345 | Q16832 | Q9UF33 | P29323 | P21860 | P11362 | P36888 | P14616 |
| P06213 | P10721 | Q12866 | P08581 | O15146 | P04629 | Q16288 | P09619 |
| P07949 | Q04912 | Q01974 | P08922 | P34925 | P35590 | Q06418 | P17948 |
| P35916 | P42684 | Q07912 | Q9UIG0 | P51813 | P19525 | Q05397 | Q14289 |
| P16591 | O60674 | Q05655 | P43405 | P07948 | P42679 | Q14680 | Q504Y2 |
| O43353 | Q9H3Y6 | O75716 | Q6J9G0 | Q13470 | P29597 | P30291 | P43403 |
| Q13131 | P31751 | Q96QP1 | Q86TB3 | Q96L96 | Q8NFD2 | Q13315 | Q13535 |
| O14965 | P11274 | Q9NSY1 | P15056 | Q8TDC3 | O60566 | O43683 | P33076 |
| O00311 | O14757 | O96017 | Q9UPZ9 | P68400 | O14936 | O14578 | P53355 |
| Q9Y4B6 | Q8N568 | Q9C098 | Q9BQI3 | Q9NZJ5 | Q9P2K8 | O75460 | Q76MJ5 |
| Q8IXL6 | Q14296 | O14976 | P49840 | Q96GX5 | Q8TF76 | Q9H422 | Q8NE63 |
| P57058 | O14920 | Q13418 | P51617 | Q9NWZ3 | Q9Y6M4 | P11801 | O75582 |
| Q9UK32 | Q96S38 | Q96LW2 | Q6VAB6 | O95835 | P53667 | Q6ZMQ8 | Q8IWU2 |
| Q96Q04 | Q38SD2 | Q5S007 | Q56UN5 | Q92918 | Q8IVH8 | P49137 | Q8IW41 |
| Q9P0L2 | O15021 | Q9BUB5 | P00540 | Q5VT25 | Q6DT37 | P42345 | Q8NEV4 |
| Q6ZWH5 | Q8NG66 | Q96PY6 | P51955 | P51956 | P51957 | Q6P3R8 | Q9HC98 |
| Q86SG6 | Q8TD19 | Q8IY84 | Q7Z2Y5 | O60285 | Q5VST9 | O75914 | Q9P286 |
| Q96RG2 | O15530 | Q99570 | Q86V86 | Q9BXM7 | Q99640 | P78527 | Q13523 |
| Q96S44 | Q9BRS2 | Q9BVS4 | O14730 | Q13546 | Q9Y572 | P57078 | O75116 |
| Q9Y6S9 | Q52WX2 | P0C263 | P0C264 | Q96BR1 | Q9H0K1 | Q9Y2K2 | Q9H2G2 |
| Q96Q15 | Q9NRH2 | Q15772 | P78362 | Q9UEE5 | Q86UX6 | O94804 | Q15831 |
| P49842 | Q9Y6E0 | Q9BXU1 | Q9BYT3 | Q8TDR2 | Q9NRP7 | Q9UEW8 | Q13188 |
| Q8N2I9 | Q9UL54 | Q9UHD2 | Q8WZ42 | Q86UE8 | Q59H18 | Q9UKE5 | O75962 |
| Q9BX84 | Q96QT4 | Q9BXA7 | Q6SA08 | Q5TCY1 | Q6IQ55 | Q8TAS1 | O75385 |
| Q6PHR2 | Q96C45 | Q86Y07 | Q9H4A3 | Q9Y3S1 | Q9BYP7 | Q96J92 | Q15118 |
| O14874 | Q13976 | Q13237 | Q15139 | Q02156 | P05129 | P41743 | Q16512 |
| Q16513 | P25098 | P34947 | Q96NX5 | Q13554 | Q16566 | Q96RR4 | Q32MK0 |
| Q15746 | P15735 | O00418 | P53350 | Q9NYY3 | O00444 | P21127 | Q14004 |
| Q00537 | Q9BWU1 | Q8IZL9 | Q00534 | P50613 | Q8IVW4 | O76039 | Q9UQ07 |
| Q16659 | Q13164 | P53779 | Q8TD08 | Q9UBE8 | O43283 | Q99558 | Q13233 |
| Q9NYL2 | Q99759 | Q9Y6R4 | Q99683 | O43318 | P41279 | P80192 | Q16671 |
| P27037 | Q13873 | P36894 | P37173 | P49761 | Q6XUX3 | Q13627 | Q92630 |
| Q15569 | P33981 | P36507 | P46734 | Q13163 | O14733 | Q96KB5 | Q16762 |
| Q9Y697 | O43766 | Q96EN8 | O96007 | O75648 | Q6IMI6 | O00204 | Q9NPF2 |
| Q9NRB3 | P52849 | Q99999 | Q7LGC8 | Q9NS84 | O60704 | Q9GZX3 | Q9Y661 |
| Q7LFX5 | Q8NCH0 | Q6IMI4 | P55809 | Q9HAC7 | Q96SZ6 | Q5VV42 | Q9HD40 |

**3. Hydrolases**

| O95479 | P49327 | P13995 | P30041 | P31939 | P27708 | P04180 | Q9NST1 |
| --- | --- | --- | --- | --- | --- | --- | --- |
| A6NGU5 | P36269 | Q6P531 | Q9UJ14 | Q8N0X4 | P35573 | Q9NP73 | Q8IV20 |
| P28907 | Q9Y223 | P49902 | Q96T60 | Q16875 | P63128 | P63135 | O60942 |
| P49789 | Q9UKK9 | Q6NT32 | P27169 | P19835 | Q8NCG7 | Q9Y5X9 | P54317 |
| Q6P1J6 | Q96AD5 | P41247 | Q7Z6Z6 | Q8WU67 | P04054 | P47712 | P0C869 |
| Q9UP65 | Q3MJ16 | Q9NZ20 | P14555 | Q9BZM2 | O15496 | Q9BZM1 | Q9HCN3 |
| Q9HDD0 | Q96KN8 | O60733 | Q86U10 | Q8IY17 | Q9NP80 | P08910 | P22303 |
| P38571 | Q15493 | Q8N2K0 | Q9BV23 | O95870 | Q05469 | Q99685 | Q14197 |
| Q9Y3E5 | Q86Y79 | Q6GMV3 | O95336 | Q15102 | Q13093 | Q9HAT2 | P10768 |
| Q6PIU2 | P28039 | Q9Y570 | Q9NUJ1 | Q8TEA8 | Q96FN9 | Q9BTV6 | Q6P988 |
| Q9BQ69 | Q53H76 | Q9Y4D2 | O14734 | Q8WXI4 | Q9NPJ3 | P49753 | O00154 |
| Q68D91 | Q5T1C6 | Q8N1Q8 | Q6NVY1 | Q16775 | Q9NV23 | Q6PCB6 | O95372 |
| Q5VWZ2 | P50897 | P05187 | Q6ZNF0 | P13686 | Q9NPH0 | P24666 | Q9BZG2 |
| P78330 | Q92539 | Q9BQK8 | O14495 | Q5VZY2 | Q96P26 | Q9H0P0 | P21589 |
| O95861 | Q9NX62 | P35575 | Q9BUM1 | O00757 | Q9UNH5 | Q16667 | O95476 |
| Q9BRF8 | Q9Y5B0 | O15194 | Q9Y6W6 | Q9UNI6 | O95147 | Q9H1R2 | Q9BY84 |
| Q8WTR2 | Q9BVJ7 | Q4G0W2 | Q68J44 | Q13115 | Q16829 | O95278 | Q92562 |
| Q9H0C8 | Q96HS1 | O60346 | P62136 | P16298 | O14829 | O14830 | O75688 |
| O15297 | Q8WY54 | O15355 | Q9ULR3 | Q8N3J5 | Q5SGD2 | Q8N819 | P53041 |
| Q8NI37 | P60484 | Q8WUK0 | Q8IXW5 | Q76I76 | Q8TE77 | Q9NP77 | Q8WVY7 |
| A6NDG6 | O14732 | Q9Y2H2 | Q8TBE9 | P32019 | Q9NRR6 | O43426 | Q9P0J1 |
| Q9NQ88 | O75061 | O95677 | Q86V88 | P30305 | P30307 | Q13615 | Q05209 |
| Q12923 | Q15678 | Q99952 | P18031 | Q4JDL3 | Q9Y2R2 | Q9H3S7 | P29074 |
| P29350 | P43378 | Q92932 | P23467 | P08575 | Q9HD43 | Q12913 | Q15262 |
| P28827 | Q16827 | Q9UMZ3 | Q15256 | Q13332 | P23471 | Q63HR2 | Q93096 |
| P56180 | Q8TF42 | Q14642 | Q15735 | P49441 | Q9UNW1 | Q13613 | Q96EF0 |
| Q9NTJ5 | Q96PE3 | O15327 | Q8TCT1 | P34913 | Q9UHY7 | Q86T03 | O15357 |
| Q7Z4H3 | Q08623 | Q9UK39 | P22413 | Q9Y2M0 | Q8WTR4 | Q9NPB8 | P17405 |
| Q13393 | O14939 | Q9NQ66 | Q01970 | Q15147 | Q8N3E9 | Q9P212 | P19174 |
| Q4KWH8 | Q6UWV6 | Q9NY59 | Q9NXE4 | O60906 | Q9Y233 | Q14123 | O00408 |
| Q14432 | P18545 | Q9HCR9 | O76074 | P16499 | O76083 | P09543 | Q6UWR7 |
| Q9NZC3 | Q9UK23 | P80108 | P27815 | Q13946 | O95263 | Q6IQ20 | P54793 |
| Q96EG1 | Q6UWY0 | P34059 | P15289 | P15848 | P22304 | P15586 | Q8N4P3 |
| Q9NVH0 | P27695 | Q9UBZ4 | O60671 | Q99638 | Q9NSU2 | Q96AZ6 | Q9ULM6 |
| Q9UFF9 | Q504Q3 | O95453 | Q6L8Q7 | Q8NA58 | Q96BZ4 | P24855 | O00115 |
| Q8WZ79 | Q9UPY3 | Q9NRR4 | O60930 | O75792 | O15091 | Q9H777 | Q9BQ52 |
| Q9H9G7 | Q7KZF4 | P0DUB6 | Q9BZP6 | Q96QH8 | Q99519 | Q8WWR8 | O43451 |
| P06280 | P16278 | O00754 | Q9Y2E5 | Q9NTJ4 | O00462 | O43280 | P08236 |
| Q9UEF7 | Q9UHN6 | Q8WUJ3 | Q12891 | P38567 | Q9HCG7 | P04062 | P54803 |
| P54802 | Q9BTY2 | P07686 | Q8WVB3 | P09848 | P35475 | Q8NFI3 | Q13724 |
| Q32M88 | Q9BZQ6 | P33908 | Q9UKM7 | P49641 | Q5SRI9 | Q9NX46 | Q86W56 |
| Q9Y251 | O60502 | Q14697 | Q10588 | O95256 | Q13478 | Q9NPH3 | Q01638 |
| Q9HB29 | Q9NZN1 | Q6SZW1 | P54922 | P29372 | P13051 | Q13569 | Q9UIF7 |
| Q96HN2 | Q8N661 | P09960 | P07099 | Q9H6B9 | P28838 | P15144 | Q9UIQ6 |
| Q07075 | O43895 | Q9NQH7 | P55786 | P53582 | P50579 | Q9HAU8 | Q9ULA0 |
| Q8IYS1 | P12955 | Q9H4A9 | Q96KN2 | P53634 | Q9NY33 | P27487 | Q6V1X1 |
| O14773 | P29144 | P12821 | P42785 | P10619 | P15085 | P15088 | P15169 |
| Q7L8A9 | Q96IY4 | Q04609 | O75976 | Q9BYF1 | Q9UPW5 | Q96MI9 | Q8NDL9 |
| Q5VU57 | Q9UBR2 | P13798 | Q9NXJ5 | Q7L266 | Q9UKU6 | Q92820 | P54252 |
| Q92560 | Q9NQC7 | Q9BSY9 | O00303 | Q15040 | Q8NBR6 | Q9H8M7 | Q4G0A6 |
| Q5VVQ6 | Q8N6M0 | Q8TE49 | Q96FW1 | Q5VV17 | Q5T2D3 | Q01804 | Q96G74 |
| Q96BN8 | P21580 | Q14694 | P51784 | O75317 | Q92995 | P54578 | Q9Y4E8 |
| Q9Y5T5 | O94966 | O94782 | Q9UK80 | Q9UPU5 | Q9BXU7 | Q96RU2 | O75604 |
| Q70CQ3 | Q70CQ4 | Q8NFA0 | Q8TEY7 | Q70CQ2 | Q9P2H5 | Q9P275 | Q86T82 |
| Q8NB14 | Q9Y6I4 | Q9NVE5 | Q3LFD5 | Q9H9J4 | Q70EL4 | Q9H0E7 | Q70EL2 |
| Q96K76 | Q86UV5 | Q70EK9 | Q93009 | P40818 | P15374 | Q9Y5K5 | O00507 |
| Q96JH7 | Q9UGI0 | Q96AP4 | Q8IYP2 | P35030 | P00734 | P00742 | P00747 |
| P98073 | P10323 | P08311 | P08709 | P48147 | P03952 | P08246 | P00748 |
| P00736 | P09871 | P05156 | P00746 | P00751 | P36776 | Q86WA8 | Q15661 |
| P00750 | P04070 | P09093 | P00749 | P09958 | P12544 | Q9BY50 | Q16740 |
| O00187 | Q9H300 | O75783 | Q9NX52 | Q8TEB9 | P05981 | O43464 | Q9Y5Y6 |
| Q14703 | P49862 | P07858 | P09668 | Q99538 | Q13867 | Q9UBX1 | P43234 |
| O60911 | Q14674 | P07384 | P20807 | P42575 | P51878 | P55212 | P55210 |
| Q14790 | P55211 | Q92851 | P07339 | Q9Y5Z0 | Q495T6 | O95450 | Q9BYT8 |
| P08254 | Q16819 | P13497 | P14780 | P14735 | Q99797 | O43847 | O75439 |
| P0DPD8 | Q13219 | P50281 | O14672 | O75173 | O75844 | O43462 | P78536 |
| Q76LX8 | A5LHX3 | P28072 | Q99436 | O94925 | Q9NQR4 | Q9UBR1 | Q63HM1 |
| P43251 | Q03154 | Q6GTS8 | Q8TDN7 | Q5QJU3 | Q9NUN7 | Q13510 | Q9NR71 |
| Q02083 | Q9Y303 | P20933 | Q96PD5 | Q969S8 | Q96IV0 | Q9HBH1 | Q9Y2B2 |
| O95498 | Q96DB2 | Q92769 | Q9UQL6 | Q9UBN7 | O00519 | Q6GMR7 | Q96HD9 |
| Q96AB6 | Q96HA8 | Q99497 | Q86X76 | Q14117 | Q6PJP8 | Q9H816 | Q96NU7 |
| O14841 | P78540 | Q9BSE5 | Q9Y2J8 | Q6TGC4 | O94760 | Q9Y2T3 | Q9NZK5 |
| P00813 | Q9BWV3 | P32320 | Q01433 | P32321 | P30793 | Q7Z6V5 | Q9BUB4 |
| P41238 | Q9Y235 | P55265 | P78563 | P31941 | Q96AK3 | Q6NTF7 | P46926 |
| P52758 | Q9H2U2 | Q9H008 | Q86TP1 | P49961 | O75355 | Q8WVQ1 | Q9Y227 |
| O75354 | P07311 | O95671 | Q9BY32 | Q9NV35 | Q9H773 | Q3LIE5 | Q9BW91 |
| Q9UHI6 | Q9BSD7 | P50583 | Q9BQG2 | Q86X67 | P33316 | Q9BU02 | Q9Y6X5 |
| Q86YN1 | O95848 | Q9BRQ3 | Q9NZJ9 | P36639 | Q6ZVK8 | Q96C86 | Q9NPI6 |
| Q8IZD4 | Q8IU60 | Q96DE0 | Q8IY26 | Q7Z2E3 | P46459 | P55072 | O75351 |
| P11021 | P07900 | Q9BVQ7 | Q8NB90 | Q9Y4B4 | Q8N3C0 | P46100 | P54132 |
| Q86WJ1 | O14647 | Q12873 | Q9P2D1 | Q96FC9 | Q9H2U1 | P51530 | Q2NKX8 |
| P18074 | P19447 | Q8NFZ0 | Q13283 | Q8NG08 | Q8TDG4 | A2PYH4 | P25205 |
| P33991 | P33992 | Q14566 | P33993 | Q9UJA3 | Q9NXL9 | Q8IVL1 | Q96RR1 |
| Q9H611 | Q92698 | P46063 | O94761 | O94762 | Q92900 | Q9NZ71 | Q9Y230 |
| P38935 | Q9H4L7 | Q14191 | O60306 | Q13206 | Q92841 | Q9NVP1 | Q92499 |
| Q9NR30 | Q9BUQ8 | Q9GZR7 | Q9UHL0 | Q96GQ7 | Q9NUL7 | Q9H8H2 | Q9UJV9 |
| Q86XP3 | Q9NXZ2 | Q7L014 | Q9Y6V7 | Q9NQI0 | Q8N8A6 | Q9Y2R4 | Q8TDD1 |
| Q8NHQ9 | Q9NY93 | O95786 | Q5T1V6 | Q8IY21 | P26196 | Q7Z478 | Q7L2E3 |
| Q7L7V1 | Q14147 | Q8IY37 | Q8IX18 | Q6P158 | Q96C10 | Q14562 | Q08211 |
| Q13838 | Q9BX63 | Q8IYD8 | P38919 | Q9BYX4 | Q9BXT6 | Q9HCE1 | P42285 |
| Q92620 | Q8IYB8 | Q587J7 | Q8NDG6 | O75643 | Q9H6S0 | Q9NVJ2 | P20338 |
| P51148 | P51149 | Q7Z444 | Q9NYN1 | P51159 | Q9H628 | P61586 | Q92963 |
| Q92737 | Q9BPW5 | Q2VIR3 | O60841 | P50570 | O60313 | P16930 | Q16719 |
| Q6P587 | Q9NRX4 | P51688 |  |  |  |  |  |

**4. Lyases**

| Q08426 | P49327 | P40939 | P51659 | Q13686 | P05177 | Q16678 | P19021 |
| --- | --- | --- | --- | --- | --- | --- | --- |
| O95954 | Q8N0X4 | P11172 | Q6YP21 | Q3LXA3 | Q6P587 | A6NK06 | O95822 |
| Q99259 | P11926 | P22234 | P19113 | Q16822 | P53602 | Q8NBZ7 | Q96CD2 |
| P06132 | Q8TDX5 | P17707 | Q9UG56 | P30046 | P38435 | Q9NTX5 | A6NGE7 |
| Q9Y315 | P04075 | O95470 | Q9UJ83 | Q9BXD5 | Q8TB92 | Q86XE5 | Q9NV66 |
| Q9NZB8 | O43570 | P22748 | Q9Y2D0 | P23280 | Q16790 | P07954 | P21399 |
| Q99798 | A6NNW6 | P06733 | P30084 | Q13825 | P35520 | P13716 | O95455 |
| O60547 | Q7Z3D6 | Q96N76 | P86397 | Q7L5Y1 | P10746 | Q96EM0 | Q8IW45 |
| Q9H0N5 | Q96GX9 | B0YJ81 | Q9P035 | Q96SQ9 | Q9BYJ1 | P24557 | Q8TBG4 |
| P60174 | Q03393 | Q96FI4 | Q969S2 | Q8TAT5 | P78549 | O15527 | P23396 |
| P42357 | Q96GA7 | Q9GZT4 | P04424 | P30566 | Q9BUX1 | Q9BVM4 | O75223 |
| P32929 | Q04760 | Q96I15 | P53701 | Q16873 | O14880 | Q08828 | Q08462 |
| O60266 | O95622 | P40145 | O60503 | Q96PN6 | P16066 | P33402 | Q02153 |
| O75343 | P25092 | P51841 | Q8NCE0 | Q9BSV6 | P07998 | P10153 | O00584 |
| P22830 |  |  |  |  |  |  |  |

**5. Isomerases**

| Q08426 | P14060 | P51589 | O60760 | O43708 | O14684 | P30084 | Q96SQ9 |
| --- | --- | --- | --- | --- | --- | --- | --- |
| Q9BYJ1 | P24557 | P60174 | Q9GZT4 | Q2QD12 | Q14376 | Q96C23 | P51606 |
| O94923 | Q9UL01 | A2VDF0 | Q96PE7 | Q9UHK6 | Q8NCW5 | Q96AY3 | Q9NYL4 |
| Q00688 | Q02790 | Q9Y680 | Q14318 | P30414 | Q13526 | Q9Y237 | Q08752 |
| Q9UNP9 | Q13427 | Q8WUA2 | Q96BP3 | Q15257 | P49247 | P34949 | P06744 |
| Q5T013 | Q9BV20 | P14174 | Q13907 | Q15125 | P42126 | O75521 | P40126 |
| Q96HD1 | Q13087 | P13667 | Q14554 | Q15084 | Q96JJ7 | Q8NBS9 | P41222 |
| Q9H7Z7 | Q15185 | P36871 | O95394 | P07738 | Q92871 | P22033 | P48449 |
| Q9Y606 | Q3MIT2 | Q8WWH5 | O95900 | Q9BZE2 | Q9NPH2 | Q8IYT4 | O75449 |
| Q9UBP0 | P13569 | P10809 | P11387 | Q13472 | O95985 | Q9Y5K1 | Q02880 |

**6. Ligases**

| P22102 | P27708 | P15104 | Q14258 | P22234 | P54577 | Q9Y2Z4 | P23381 |
| --- | --- | --- | --- | --- | --- | --- | --- |
| Q9UGM6 | A2RTX5 | Q9P2J5 | Q15031 | P41252 | Q9NSE4 | Q15046 | Q5JTZ9 |
| P26640 | P56192 | Q96GW9 | P49591 | Q9NP81 | P14868 | Q6PI48 | P41250 |
| P07814 | Q7L3T8 | P49589 | Q9HA77 | Q5JPH6 | P47897 | P54136 | Q5T160 |
| Q9Y285 | Q9NSD9 | O95363 | P12081 | O43776 | Q96I59 | Q9NUB1 | Q9NR19 |
| Q9H6R3 | Q14689 | Q96CM8 | O95573 | Q53FZ2 | Q96GR2 | P33121 | Q6PCB7 |
| Q9Y2P5 | P53597 | Q9P2R7 | Q86V21 | P22314 | Q8TBC4 | Q4G176 | Q7L8W6 |
| P48506 | P48637 | A5YM72 | Q05932 | Q8NG68 | Q8IXN7 | Q9HAB8 | P49914 |
| P17812 | Q6UB35 | Q8N142 | P00966 | P50747 | Q6XQN6 | Q6IA69 | P49915 |
| O15067 | P08243 | Q9H0R6 | P11498 | O00763 | P05165 | P05166 | Q96RQ3 |
| Q9HCC0 | P18858 | P49916 | P49917 | O00442 | Q9Y3I0 |  |  |

**7. Translocases**

| Q13423 | P28331 | O75306 | O75489 | O75251 | O00217 | P49821 | P19404 |
| --- | --- | --- | --- | --- | --- | --- | --- |
| P03886 | P03891 | P03897 | P03905 | P03915 | P03923 | P08574 | P47985 |
| P00395 | P00403 | P00414 | P06576 | P38606 | Q8N8Q1 | Q53TN4 | Q04656 |
| P16615 | Q01814 | O75185 | P54707 | Q9NP78 | Q99758 | P78363 | Q8IZY2 |
| Q86UK0 | O60312 | Q9Y2G3 | Q9NTI2 | P98198 | O60423 | O43861 | P21439 |
| Q9UNQ0 | Q92887 | O15439 | O15440 | Q5T3U5 | Q96J66 | Q15311 | O95255 |
| Q9NP58 | O14678 | Q9H7F0 |  |  |  |  |  |
