# Supplementary material for: PredictEFC: a fast and efficient multi-label classifier for predicting enzyme family classes
Source: BMC Bioinformatics. 2024 Jan 30;25:50. doi: 10.1186/s12859-024-05665-1 (PMC10829269; doi:10.1186/s12859-024-05665-1)
Supplement: Supplementary file 2 — Additional file 2. Independent dataset retrieved from Expasy [file 12859_2024_5665_MOESM2_ESM.docx]

**Additional file 2.** Independent dataset retrieved from Expasy.

1. **Oxidoreductases**

| Q9UKU7 | Q8IUF8 | Q6V0L0 | A0A087X1C5 | Q687X5 |  |  |  |
| --- | --- | --- | --- | --- | --- | --- | --- |

1. **Transferases**

| Q96MH6 | Q9BTE0 | O60683 | P28328 | Q9H920 | Q8N5U6 | Q9C019 | Q9H8W5 |
| --- | --- | --- | --- | --- | --- | --- | --- |
| Q8IWR1 | Q6PJ69 | Q96S15 | Q9Y5Z9 | P49842 |  |  |  |

**3. Hydrolases**

| A0A1W2PQ27 | Q8IWU6 | Q8N2M4 | Q9UMW8 | Q53GS9 | O15393 | A0A024RBG1 | P49736 |
| --- | --- | --- | --- | --- | --- | --- | --- |
| Q15477 | P61011 | Q9NP77 | Q9UHN6 | Q9NZJ9 | Q86WJ1 |  |  |

**4. Lyases**

**5. Isomerases**

**6. Ligases**

**7. Translocases**

| P03901 | Q03518 |  |  |  |  |  |  |
| --- | --- | --- | --- | --- | --- | --- | --- |
