# Supplementary material for: PredictEFC: a fast and efficient multi-label classifier for predicting enzyme family classes
Source: BMC Bioinformatics. 2024 Jan 30;25:50. doi: 10.1186/s12859-024-05665-1 (PMC10829269; doi:10.1186/s12859-024-05665-1)
Supplement: Supplementary file 3 — Additional file 3. Independent dataset retrieved from KEGG ENZYME [file 12859_2024_5665_MOESM3_ESM.docx]

**Additional file 3.** Independent dataset retrieved from KEGG ENZYME.

1. **Oxidoreductases**
2. **Transferases**

| O43709 | Q1RMZ1 | Q9Y5N5 | Q7Z4G4 | Q96P11 | P46087 | Q9UI43 | O43159 |
| --- | --- | --- | --- | --- | --- | --- | --- |
| Q9HBH9 | Q86YV5 | Q8IV63 | Q13308 | Q9BUB5 | O43187 | Q9Y616 | Q9HA64 |
| Q8NB16 | Q9H792 | Q9H479 | P07332 | P16591 | Q9H0J9 | Q7Z2W4 | Q460N5 |
| Q9NR21 | Q7Z3E1 | Q8N5Y8 | Q53GL7 | Q8IXQ6 | Q9UKK3 | Q8N3A8 | Q9Y6F1 |
| Q460N3 | Q9NXA8 | Q8IZD2 | Q8N8M0 | Q9H9T3 | Q9BTE0 | Q13395 | Q7Z460 |
| P08263 | Q7RTV2 | P0CG30 | Q9Y6F7 | Q9Y232 | Q16762 | P25325 | Q96LW4 |
| Q5I7T1 | Q5BKT4 | O75340 | Q9NZL9 | A6NG13 | P00709 | O76075 | Q96IJ6 |
| Q9Y274 |  |  |  |  |  |  |  |

1. **Hydrolases**

| Q9P0U3 | Q9HC62 | Q9BQF6 | Q9H4L4 | Q96LD8 | Q9UMW8 | Q96HI0 | Q9GZR1 |
| --- | --- | --- | --- | --- | --- | --- | --- |
| P20618 | P28066 | P49721 | P49720 | P28070 | P60900 | Q8TAA3 | P25788 |
| P25789 | P25786 | Q05315 | A1Z1Q3 | Q9BQ69 | Q9UQQ1 | Q58DX5 | P14621 |
| P07311 | Q96DG6 | Q9UMR5 | Q8N4T0 | P15085 | Q8WXQ8 | Q96IY4 | Q8NE79 |
| O94760 | O95865 | Q2TAA2 | O43681 | Q15477 | O75319 | Q8NCE2 | Q5VZP5 |
| Q93096 | O75365 | Q96FJ0 | Q9H4B8 | Q9H4A9 | Q9UKR3 | P49862 | O15393 |
| P0DTE8 | P0DUB6 | A8MYZ0 | Q9BZM1 | Q9BX93 | Q5R387 | Q9NPB1 | Q99988 |
| P57075 | Q7L5L3 | O94759 | Q10713 |  |  |  |  |

**4. Lyases**

| P30046 | A6NHG4 | P35219 | P09104 | P06733 |  |  |  |
| --- | --- | --- | --- | --- | --- | --- | --- |

**5. Isomerases**

| Q96PZ0 | Q9H0K6 | O75344 | Q6UX04 | Q8IXY8 | Q96AT9 | Q13011 | Q8IZU8 |
| --- | --- | --- | --- | --- | --- | --- | --- |

**6. Ligases**

| Q9UBT2 | Q9UBE0 | Q3SXZ7 | O95922 | Q6EMB2 | Q8N841 |  |  |
| --- | --- | --- | --- | --- | --- | --- | --- |

**7. Translocases**

| Q03518 | P03901 | P49447 |  |  |  |  |  |
| --- | --- | --- | --- | --- | --- | --- | --- |
