# Supplementary material for: PredictEFC: a fast and efficient multi-label classifier for predicting enzyme family classes
Source: BMC Bioinformatics. 2024 Jan 30;25:50. doi: 10.1186/s12859-024-05665-1 (PMC10829269; doi:10.1186/s12859-024-05665-1)
Supplement: Supplementary file 4 — Additional file 4. Box plot to show the distribution of IPR terms on six family classes according to the different ranges of their distribution on a given family class [file 12859_2024_5665_MOESM4_ESM.docx]

**Additional file 4.** Box plot to show the distribution of IPR terms on six family classes according to the different ranges of their distribution on a given family class.


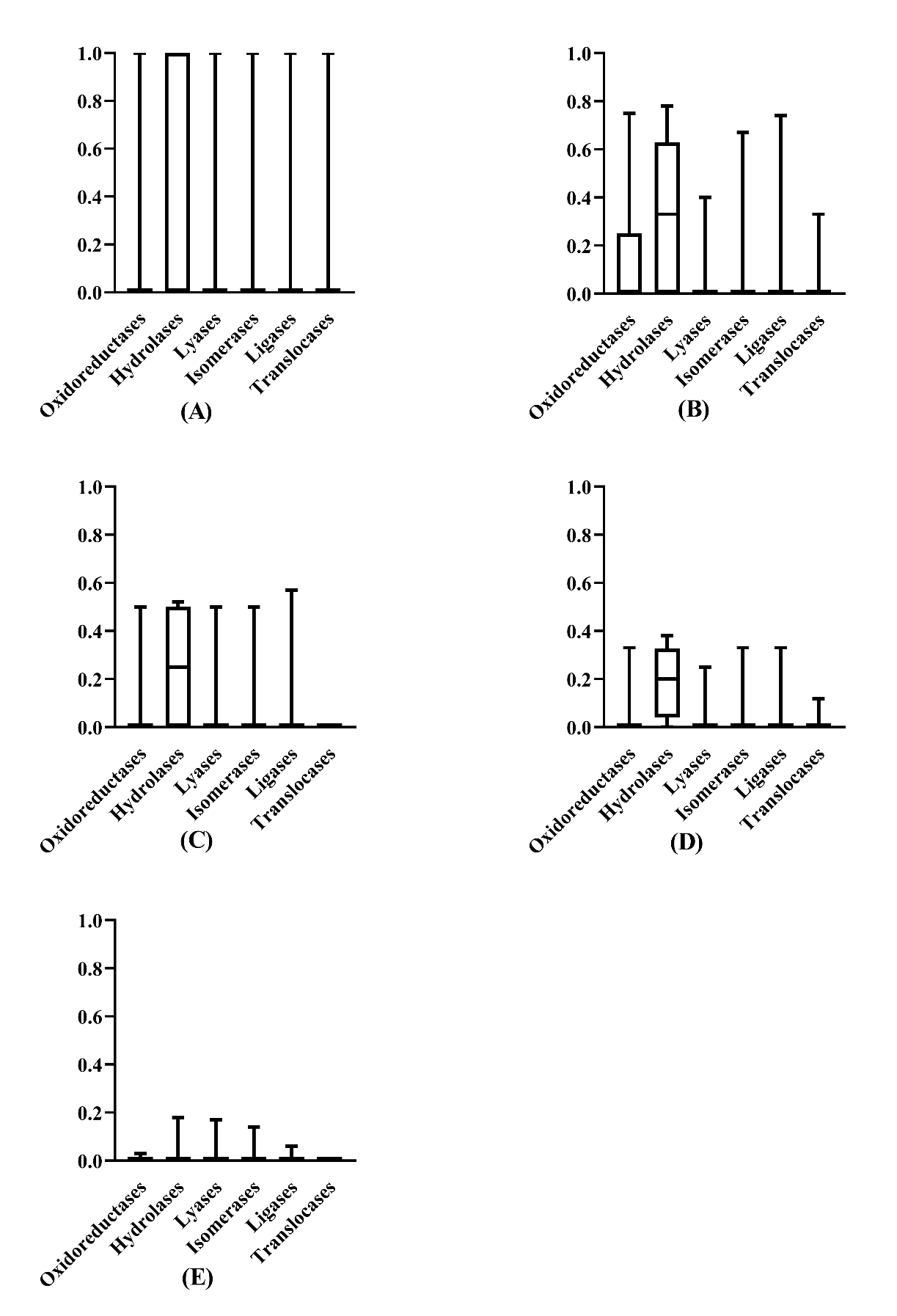


**Figure S1.** Box plot to show the distribution of IPR terms on six family classes according to the different ranges of their distribution on transferases. (A) the range is [0, 0.2]; (B) the range is (0.2, 0.4]; (C) the range is (0.4-0.6]; (D) the range is (0.6-0.8]; (E) the range is (0.8-1.0].


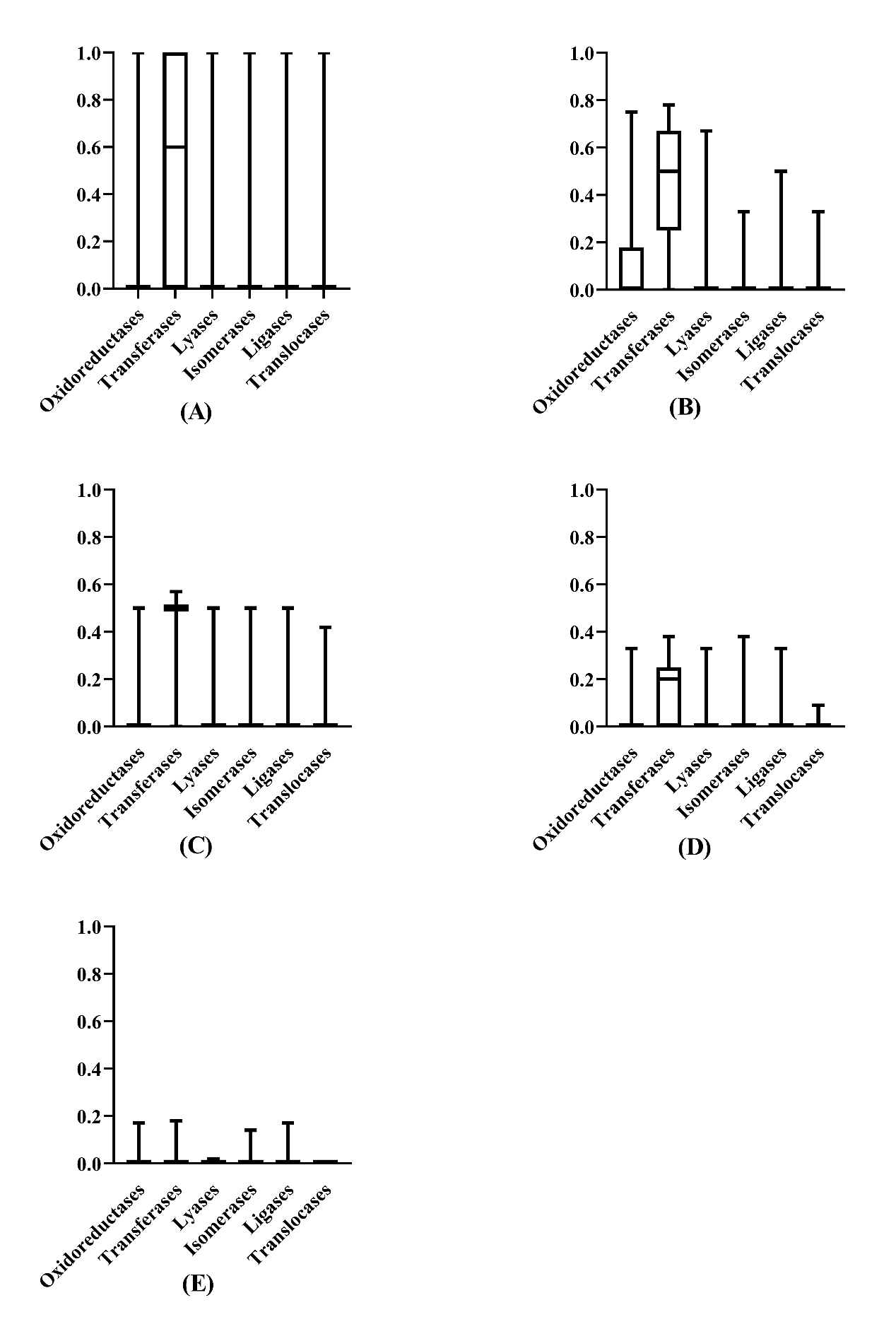


**Figure S2.** Box plot to show the distribution of IPR terms on six family classes according to the different ranges of their distribution on hydrolases. (A) the range is [0, 0.2]; (B) the range is (0.2, 0.4]; (C) the range is (0.4-0.6]; (D) the range is (0.6-0.8]; (E) the range is (0.8-1.0].


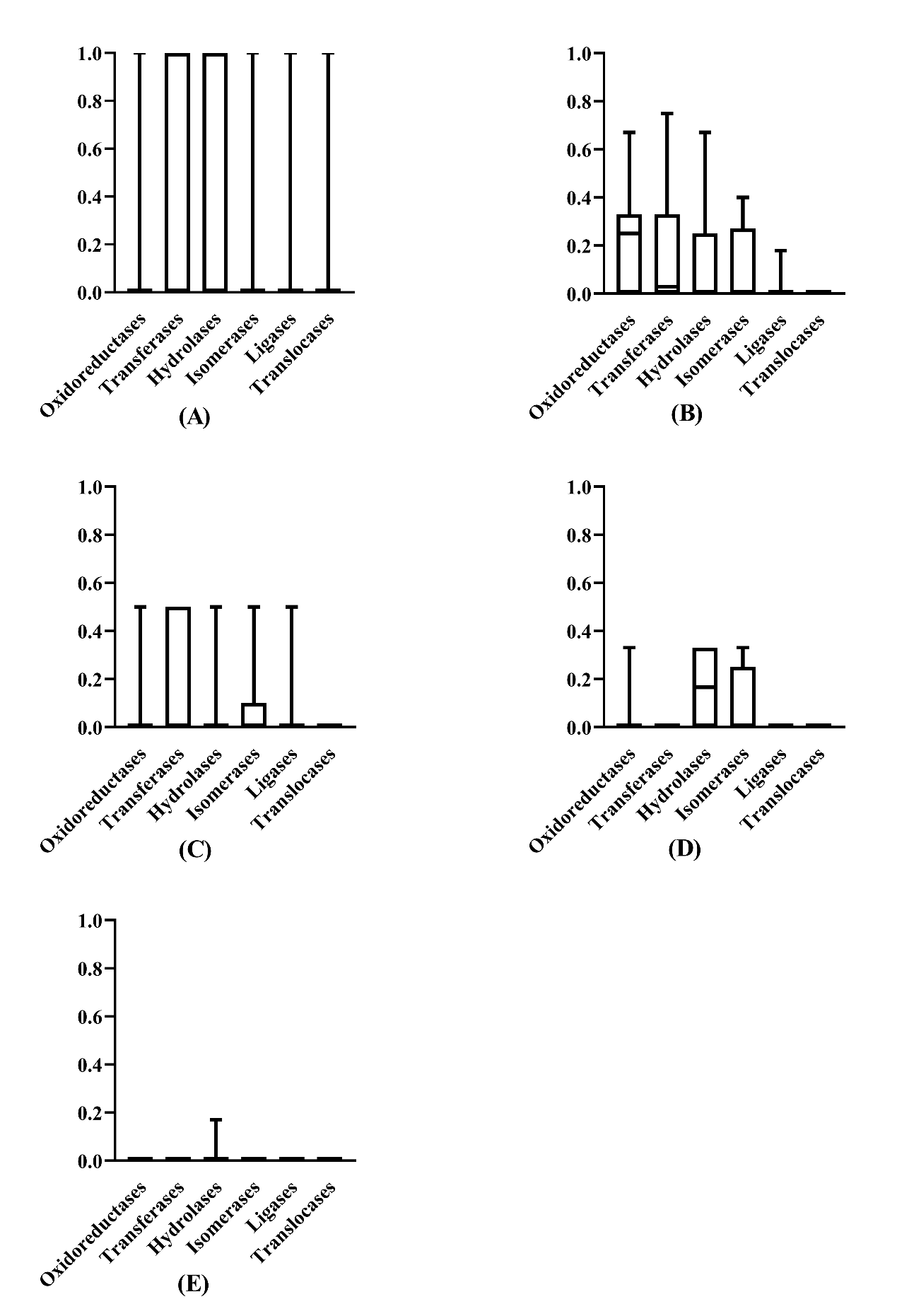


**Figure S3.** Box plot to show the distribution of IPR terms on six family classes according to the different ranges of their distribution on lyases. (A) the range is [0, 0.2]; (B) the range is (0.2, 0.4]; (C) the range is (0.4-0.6]; (D) the range is (0.6-0.8]; (E) the range is (0.8-1.0].


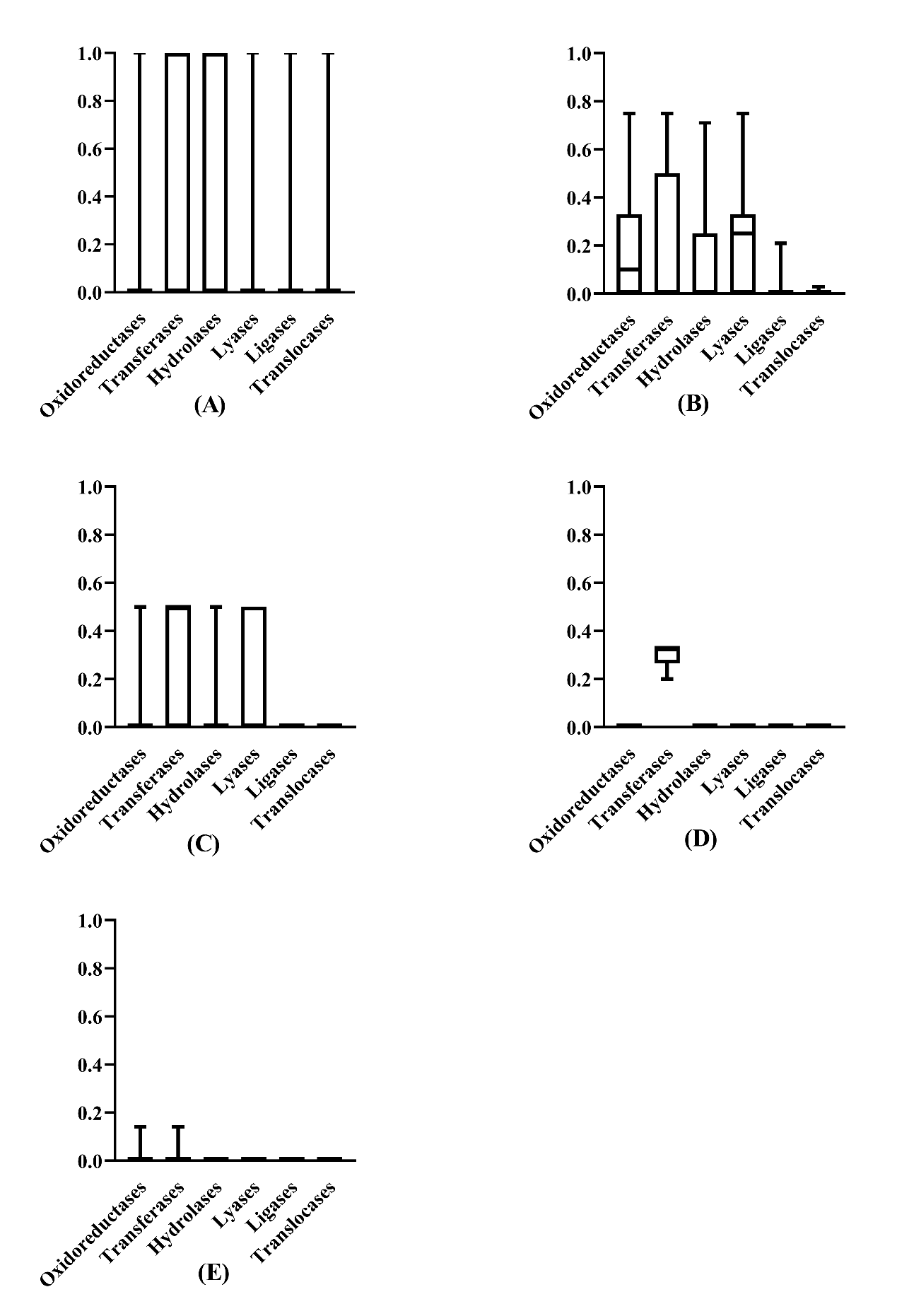


**Figure S4.** Box plot to show the distribution of IPR terms on six family classes according to the different ranges of their distribution on isomerases. (A) the range is [0, 0.2]; (B) the range is (0.2, 0.4]; (C) the range is (0.4-0.6]; (D) the range is (0.6-0.8]; (E) the range is (0.8-1.0].


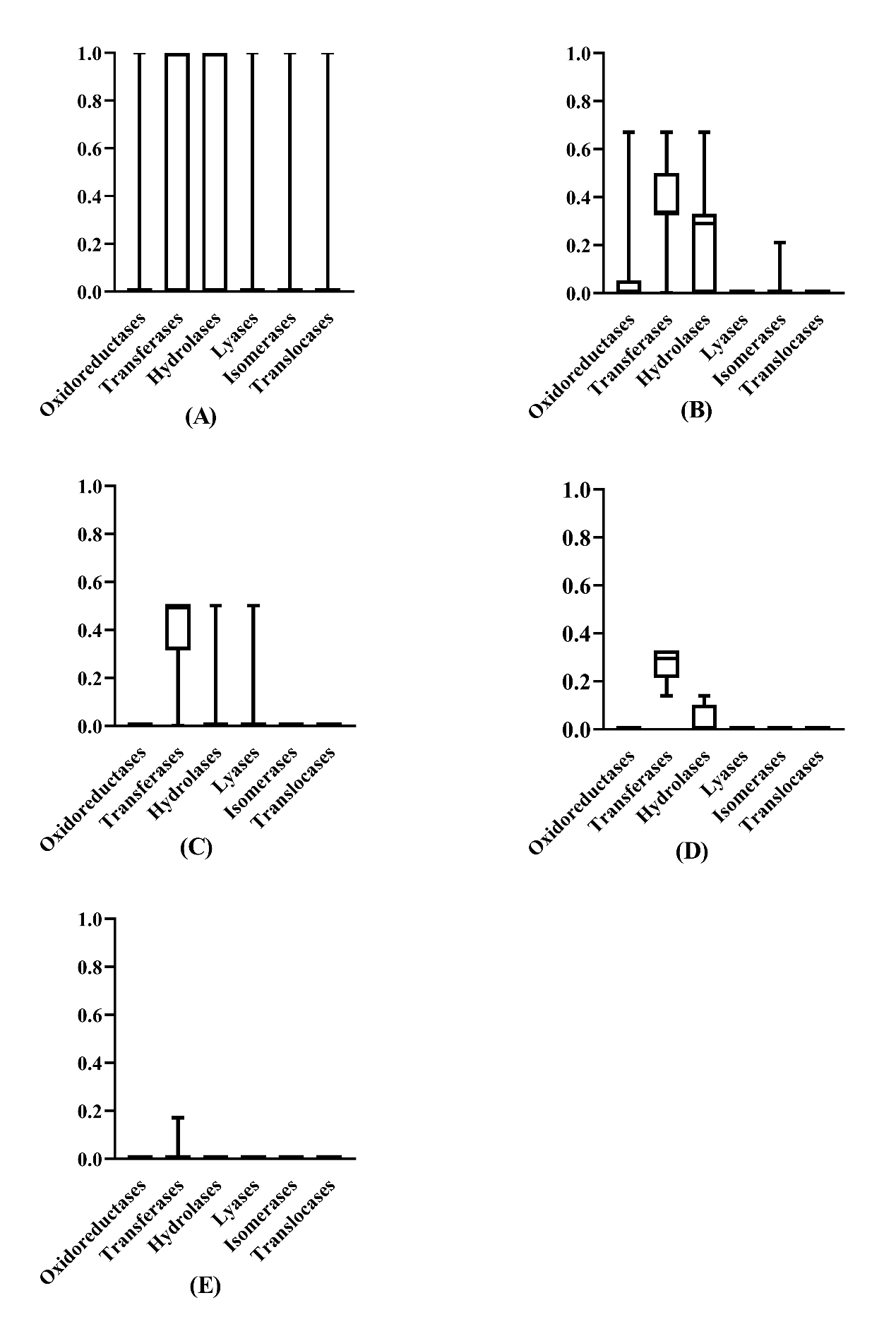


**Figure S5.** Box plot to show the distribution of IPR terms on six family classes according to the different ranges of their distribution on ligases. (A) the range is [0, 0.2]; (B) the range is (0.2, 0.4]; (C) the range is (0.4-0.6]; (D) the range is (0.6-0.8]; (E) the range is (0.8-1.0].


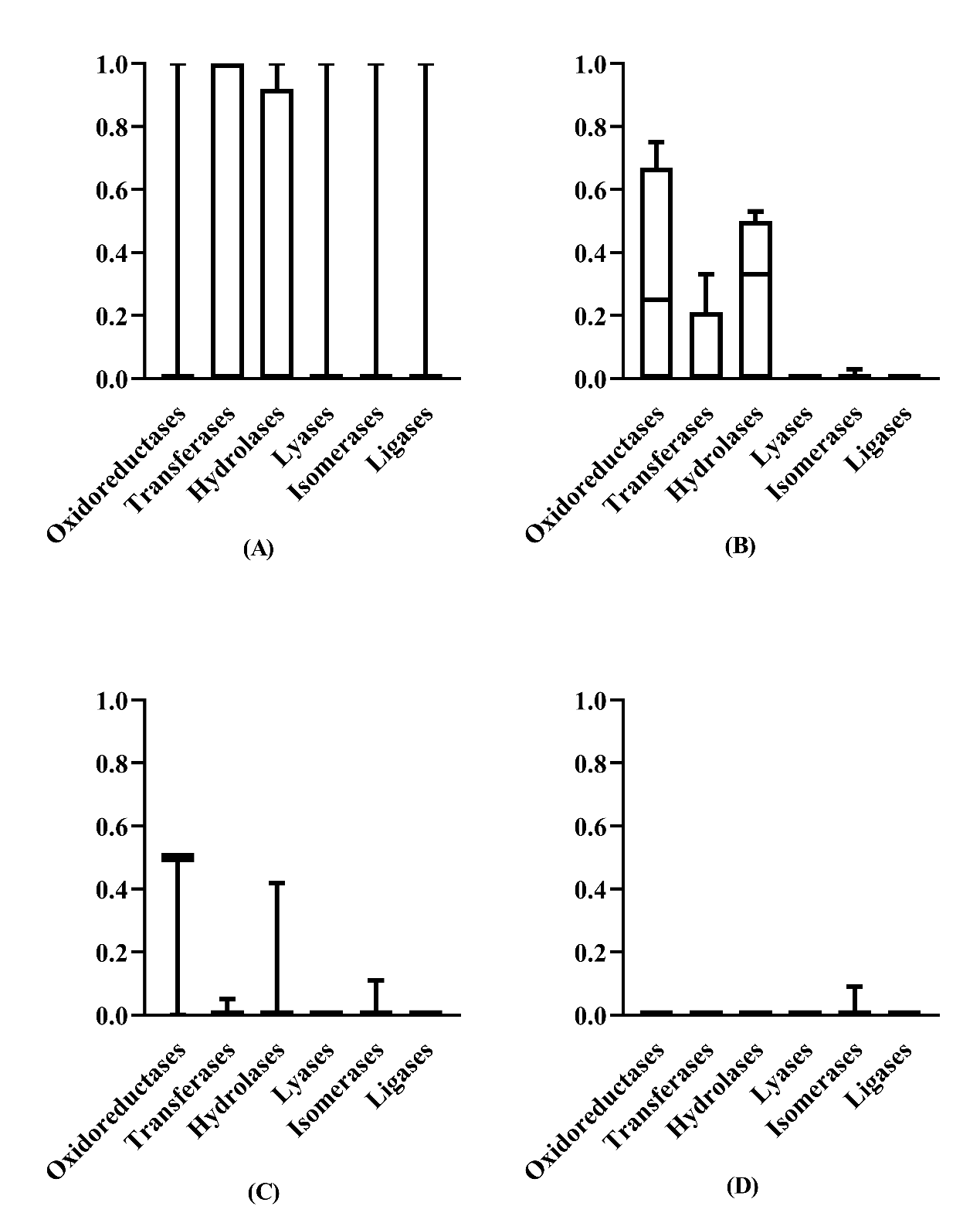


**Figure S6.** Box plot to show the distribution of IPR terms on six family classes according to the different ranges of their distribution on translocases. (A) the range is [0, 0.2]; (B) the range is (0.2, 0.4]; (C) the range is (0.4-0.6]; (D) the range is (0.8-1.0].
